# Clarithromycin expands CD11b^+^Gr-1^+^ cells via the STAT3/Bv8 axis to ameliorate lethal endotoxic shock and post-influenza bacterial pneumonia

**DOI:** 10.1371/journal.ppat.1006955

**Published:** 2018-04-05

**Authors:** Ho Namkoong, Makoto Ishii, Hideki Fujii, Kazuma Yagi, Takahiro Asami, Takanori Asakura, Shoji Suzuki, Ahmed E. Hegab, Hirofumi Kamata, Sadatomo Tasaka, Koji Atarashi, Nobuhiro Nakamoto, Satoshi Iwata, Kenya Honda, Takanori Kanai, Naoki Hasegawa, Shigeo Koyasu, Tomoko Betsuyaku

**Affiliations:** 1 Division of Pulmonary Medicine, Department of Medicine, Keio University School of Medicine, Tokyo, Japan; 2 Japan Society for the Promotion of Science, Tokyo, Japan; 3 Department of Immunology, Graduate School of Medicine, University of the Ryukyus, Okinawa, Japan; 4 Department of Microbiology and Immunology, Keio University School of Medicine, Tokyo, Japan; 5 Division of Gastroenterology and Hepatology, Department of Medicine, Keio University School of Medicine, Tokyo, Japan; 6 Center for Infectious Diseases and Infection Control, Keio University School of Medicine, Tokyo, Japan; 7 Department of Infectious Diseases, Keio University School of Medicine, Tokyo, Japan; 8 Laboratory for Immune Cell Systems, RIKEN Center for Integrative Medical Sciences, Kanagawa, Japan; Columbia University, UNITED STATES

## Abstract

Macrolides are used to treat various inflammatory diseases owing to their immunomodulatory properties; however, little is known about their precise mechanism of action. In this study, we investigated the functional significance of the expansion of myeloid-derived suppressor cell (MDSC)-like CD11b^+^Gr-1^+^ cells in response to the macrolide antibiotic clarithromycin (CAM) in mouse models of shock and post-influenza pneumococcal pneumonia as well as in humans. Intraperitoneal administration of CAM markedly expanded splenic and lung CD11b^+^Gr-1^+^ cell populations in naïve mice. Notably, CAM pretreatment enhanced survival in a mouse model of lipopolysaccharide (LPS)-induced shock. In addition, adoptive transfer of CAM-treated CD11b^+^Gr-1^+^ cells protected mice against LPS-induced lethality via increased IL-10 expression. CAM also improved survival in post-influenza, CAM-resistant pneumococcal pneumonia, with improved lung pathology as well as decreased interferon (IFN)-γ and increased IL-10 levels. Adoptive transfer of CAM-treated CD11b^+^Gr-1^+^ cells protected mice from post-influenza pneumococcal pneumonia. Further analysis revealed that the CAM-induced CD11b^+^Gr-1^+^ cell expansion was dependent on STAT3-mediated Bv8 production and may be facilitated by the presence of gut commensal microbiota. Lastly, an analysis of peripheral blood obtained from healthy volunteers following oral CAM administration showed a trend toward the expansion of human MDSC-like cells (Lineage^−^HLA-DR^−^CD11b^+^CD33^+^) with increased arginase 1 mRNA expression. Thus, CAM promoted the expansion of a unique population of immunosuppressive CD11b^+^Gr-1^+^ cells essential for the immunomodulatory properties of macrolides.

## Author summary

Myeloid-derived suppressor cells (MDSCs) are a heterogeneous population of anti-inflammatory myeloid progenitors that expand in response to acute and chronic inflammation as well as in various diseases, such as autoimmune diseases and cancer. The macrolide antibiotic clarithromycin has immunomodulatory effects in various inflammatory diseases, distinct from its antimicrobial effects, but the mechanism underlying these effects is unknown. The present study demonstrates that clarithromycin treatment induces a marked expansion of CD11b^+^Gr-1^+^ MDSC-like cells in the spleen and lungs, sufficient to protect mice from LPS-induced lethality and clarithromycin-resistant bacterial pneumonia via increased IL-10 and decreased IFN-γ levels. Clarithromycin-induced CD11b^+^Gr-1^+^ cell expansion was dependent on STAT3-mediated Bv8 production. Moreover, expansion of the immunosuppressive MDSC-like cell population was observed following clarithromycin treatment in humans. Collectively, these results suggest that the immunomodulatory effects of clarithromycin can be attributed to the induction of CD11b^+^Gr-1^+^ MDSC-like cells via the STAT3/Bv8 axis.

## Introduction

Macrolides have immunomodulatory properties in addition to their antibacterial effects [1, 2]. Indeed, previous clinical trials have shown that macrolides exhibit clinical benefits for various pulmonary diseases, including diffuse panbronchiolitis [3], chronic obstructive pulmonary disease [4], acute respiratory distress syndrome [5], cystic fibrosis [6], and non-cystic fibrosis bronchiectasis [7]. However, the precise mechanism underlying their favorable immunomodulatory effects remains largely unknown.

Previous studies of macrolides have focused on their in vitro effects on isolated cells, such as immune cells, epithelial cells, endothelial cells, and fibroblasts [8]. In particular, macrolides inhibit the production of pro-inflammatory cytokines [9]; however, the effects of macrolides on specific immune cell populations have not been examined. We hypothesized that the immunomodulatory properties of macrolides are physiological in origin, as they exhibit globally beneficial effects for various inflammatory diseases with different pathogenesis.

Myeloid-derived suppressor cells (MDSCs), a heterogeneous population of myeloid progenitors, are expanded in various diseases, including cancer, bacterial and parasitic infections, acute and chronic inflammation, sepsis, transplantation, and autoimmunity, and can potently suppress T-cell responses [10–12]. In mice, MDSCs express both CD11b and Gr-1, whereas human MDSCs mainly express Lineage^*−*^HLA-DR^−^CD11b^+^CD33^+^ or CD11b^+^CD14^−^CD33^+^, but lack markers homologous to mouse Gr-1 [10, 11]. Here, we present evidence that clarithromycin (CAM), a 14-membered macrolide, expands the immunosuppressive CD11b^+^Gr-1^+^ cell population to ameliorate lipopolysaccharide (LPS)-induced shock and post-influenza pneumococcal pneumonia in mice. We also demonstrate a CAM-induced expansion of the human Lineage^−^HLA-DR^−^CD11b^+^CD33^+^ cell population in healthy volunteers similar to that observed for CD11b^+^Gr-1^+^ cells in mice. Our findings shed new light on the novel immunomodulatory mechanism of macrolides and will contribute to the development of novel approaches to treat inflammatory diseases.

## Results

### Clarithromycin expands the CD11b^+^Gr-1^+^ cell population

**Fig 1:**
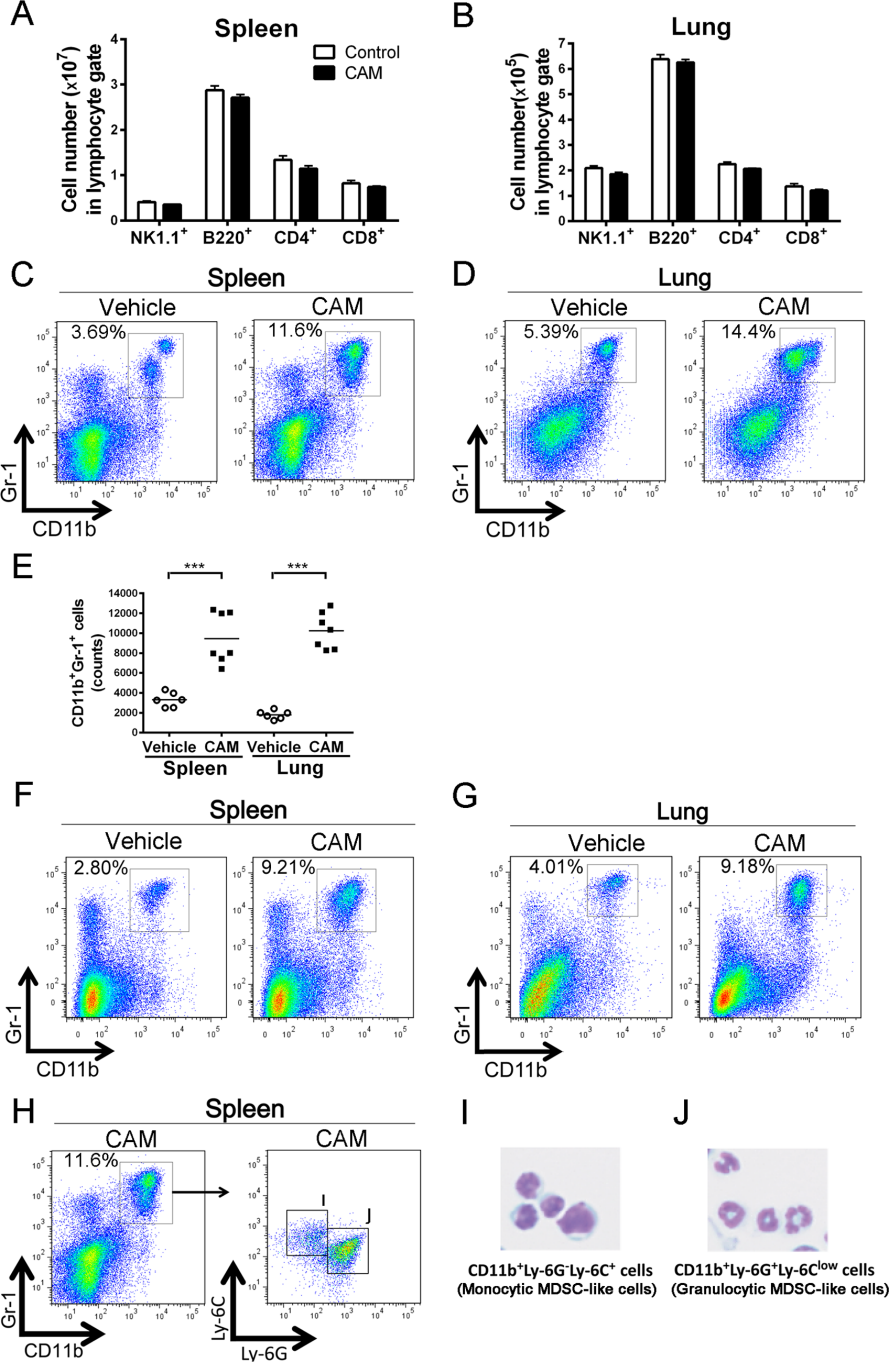
Clarithromycin expands CD11b^+^Gr-1^+^ cells in naive mice. (A and B) Quantification of major lymphocyte populations, including B220^+^, NK1.1^+^, CD4^+^, and CD8^+^ cells, in the lymphocyte gate in the spleen (A) and lungs (B) sorted from mice that were intraperitoneally treated with vehicle or clarithromycin (CAM; 100 mg/day) daily for three consecutive days. Data are expressed as the mean ± SEM (n = 4 per group). (C and D) Representative two-parameter dot plots of CD11b^+^Gr-1^+^ cells in the spleen (C) and lungs (D) sorted from mice intraperitoneally treated with vehicle or CAM daily for three consecutive days. (E) Quantification of CD11b^+^Gr-1^+^ cells in the spleen and lungs sorted from intraperitoneally vehicle- and CAM-treated mice (n = 6–7 per group). ***p < 0.001 by the Mann–Whitney U-test. (F and G) Representative two-parameter dot plots of CD11b^+^Gr-1^+^ cells in the spleen (F) and lungs (G) sorted from mice intragastrically administered vehicle or CAM (100 mg/day) daily for 14 consecutive days (n = 4 per group). (H) CAM-treated CD11b^+^Gr-1^+^ cells in the spleen were divided into two groups: CD11b^+^Ly-6G^−^Ly-6C^+^ cells and CD11b^+^Ly-6G^+^Ly-6C^low^ cells (n = 4 per group). (I and J) Representative cytospin images of (I) CD11b^+^Ly-6G^−^Ly-6C^+^ cells (monocytic MDSC-like cells) and (J) CD11b^+^Ly-6G^+^Ly-6C^low^ cells (granulocytic MDSC-like cells) (n = 4 per group).

We first examined whether macrolides, in this case CAM, could modulate immune cell populations in wild-type (WT) mice. Naive WT mice were intraperitoneally administered CAM daily for 3 days, and single-cell suspensions prepared from the spleen and lungs were analyzed by flow cytometry on the day after the last injection. We did not detect significant differences in the numbers of NK1.1^+^, B220^+^, CD4^+^, and CD8^+^ lymphocytes in both the spleen and lungs between vehicle- and CAM-treated mice (Fig 1A and 1B). Conversely, CAM administration markedly expanded the splenic and lung CD11b^+^Gr-1^+^ cell population in naive mice (Fig 1C–1E). This significant increase in CD11b^+^Gr-1^+^ cell number was also observed after oral administration of CAM for 2 weeks (Fig 1F and 1G). Next, we intraperitoneally injected CAM at various doses daily for three consecutive days and analyzed cell expansion 24 h after the last injection. As expected, CAM administration increased the CD11b^+^Gr-1^+^ cell population in a dose-dependent and time-dependent manner (S1 Fig). We further analyzed two populations of Gr-1^+^ cells, i.e., the Ly-6G^+^ and Ly-6C^+^ cell populations. Indeed, CAM-treated CD11b^+^Gr1^+^ cells were divided into two subsets: CD11b^+^Ly-6G^−^Ly-6C^+^ cells and CD11b^+^Ly-6G^+^Ly-6C^low^ cells (Fig 1H). A cytospin analysis revealed that CD11b^+^Ly-6G^*−*^Ly-6C^+^ cells displayed a monocytic morphology (Fig 1I), whereas CD11b^+^Ly-6G^+^Ly-6C^low^ cells showed a granulocytic morphology (Fig 1J).

The lungs were harvested from mice administered CAM daily for three consecutive days 24 h after the last CAM injection and stained with Gr-1 antibody. Of note, only Gr-1^+^ immunostaining was used because a previous flow cytometry analysis revealed that nearly all Gr-1^+^ cells in the lungs after CAM treatment were CD11b^+^ cells (Fig 1D). Interestingly, Gr-1^+^ cells increased markedly after CAM treatment and were mainly located in the extravascular space of the lungs (i.e., the lung parenchyma or interstitium) (S2 Fig).

### CAM-treated CD11b^+^Gr-1^+^ cell population exhibits immunomodulatory properties

**Fig 2:**
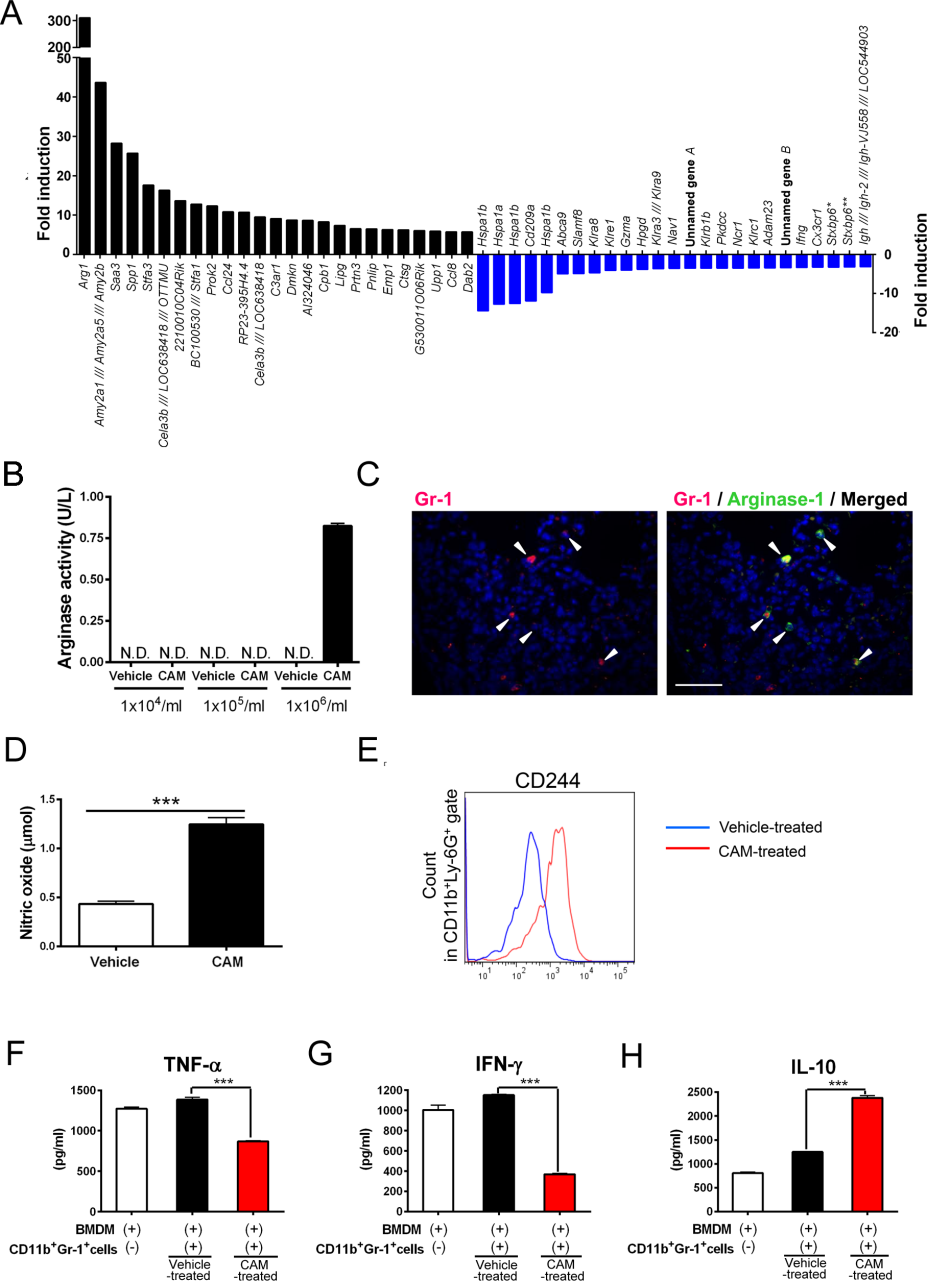
CAM-treated CD11b^+^Gr-1^+^ cells exhibit an immunosuppressive phenotype. (A) Top 25 upregulated and downregulated genes determined by a microarray analysis in splenic CD11b^+^Gr-1^+^ cells sorted from vehicle- and CAM-treated mice. *Stxbp6 (chromosome 12:45956210–46175345). **Stxbp6 (chromosome 12:45953470–45956090). Results are presented as fold changes relative to the expression levels of each gene in vehicle-treated CD11b^+^Gr-1^+^ cells. (B) Arginase activity in the spleen of vehicle- and CAM-treated mice (n = 4 per group). N.D., not detected. (C) Immunofluorescence staining of Gr-1 and arginase-1 in the lungs of mice treated with CAM daily for three consecutive days (n = 4 per group). Scale bar, 200 μm. (D) The concentration of nitric oxide (NO) in spleen extracts of vehicle- and CAM-treated mice (n = 4 per group) ***p < 0.001 by the Mann–Whitney U-test. (E) Expression of the surface marker CD244 on splenic CD11b^+^Ly-6G^+^ cells determined by flow cytometry (n = 4 per group). (F–H) Cytokine profile of the culture supernatant from bone marrow-derived macrophages (BMDMs) with or without equal numbers of vehicle-treated or CAM-treated CD11b^+^Gr-1^+^ cells (5 × 10^5^ cells) in the spleen: TNF-α (F), IFN-γ (G), and IL-10 (H). Representative data for three independent experiments are shown. Data are expressed as the mean ± SEM. ***p < 0.001 by a one-way ANOVA with Tukey’s multiple comparison tests.

To further characterize the CAM-treated CD11b^+^Gr-1^+^ cell phenotype, we performed a microarray analysis with sorted splenic CD11b^+^Gr-1^+^ cells obtained from vehicle- and CAM-treated mice. We detected a 310-fold increase in the expression of arginase-1 expression, which is a key enzyme inhibiting T cell proliferation and an MDSC marker (Fig 2A). We confirmed that both intraperitoneal injection and oral administration of CAM induced arginase-1 mRNA expression in splenic CD11b^+^Gr-1^+^ cells (S3 Fig). We further measured arginase activity in the spleen and arginase-1 expression in the lungs. High arginase activity was detected in CAM-treated CD11b^+^Gr-1^+^ cells, whereas no activity was detected in vehicle-treated CD11b^+^Gr-1^+^ cells (Fig 2B). In addition, immunostaining analysis confirmed that Gr-1^+^ cells produce arginase-1 in the lungs of CAM-treated mice (Fig 2C).

We next measured the concentration of splenic nitric oxide (NO), a crucial parameter for MDSC biology and identification [13]. The NO concentration was significantly higher in the spleen of CAM-treated mice than in that of vehicle-treated mice (Fig 2D).

To further investigate the function of CAM-treated CD11b^+^Gr-1^+^ cells in comparison with neutrophils and monocytes/macrophages, elastase activity, MPO activity, and phagocytic activity were measured (S4 Fig). Elastase activity in CAM-treated CD11b^+^Gr-1^+^ cells was lower than that in any other group (vehicle-treated CD11b^+^Gr-1^+^ cells [mainly steady-state non-activated neutrophils and monocytes/macrophages], LPS-treated CD11b^+^Gr-1^+^ cells (mainly activated neutrophils and monocytes/macrophages), thioglycolate-elicited sterile peritonitis-induced activated neutrophils, and isolated peripheral non-activated neutrophils) (S4A Fig), consistent with the notion that elastase activity in MDSCs is lower than that in neutrophils. MPO activity in CAM-treated CD11b^+^Gr-1^+^ cells was greater than that in vehicle-treated CD11b^+^Gr-1^+^ cells and in peripheral neutrophils, which appeared to be non-activated steady-state neutrophils or monocytes/macrophages. However, it was lower than that in LPS-treated CD11b^+^Gr-1^+^ cells (activated neutrophils and monocytes/macrophages) and thioglycolate-elicited sterile peritonitis-induced activated neutrophils (S4B Fig). In addition, phagocytic activity in CAM-treated CD11b^+^Gr-1^+^ cells was lower than that in any other group (S4C Fig), supporting the notion that CAM-treated CD11b^+^Gr-1^+^ cells are not neutrophils or monocytes/macrophages, but MDSC-like cells.

To verify that CAM-treated CD11b^+^Gr-1^+^ cells suppressed T cell activity, the minimal functional characteristic necessary to identify cells as MDSCs [13], we performed carboxyfluorescein succinimidyl ester (CFSE) T cell proliferation assays. The histogram of CD3^+^ T cells co-cultured with CAM-treated CD11b^+^Gr-1^+^ cells shifted to the right along the *X*-axis when compared to that for the vehicle-treated CD11b^+^Gr-1^+^ cells, but the degree of the shift was modest. These results indicate that CAM-treated CD11b^+^Gr-1^+^ cells might hinder CD3^+^ T cell proliferation (S5 Fig).

We next examined the CAM-treated CD11b^+^Gr-1^+^ cells for the presence of various surface markers expressed on MDSCs and/or myeloid cells, including CD244 [14], CTLA-4 [15], PD-1 [16], PD-L1 [17], CXCR2 [11], CXCR4 [11], CD80 [11], CD115 [11], and CX3CR1 [18]. There were no dramatic differences in the expression of these markers, but CD244 and CTLA-4 expression tended to be slightly higher in CAM-treated CD11b^+^Gr-1^+^ cells than in vehicle- treated CD11b^+^Gr-1^+^ cells (S6 Fig). Because CD244 is specifically expressed on granulocytic MDSCs [14], we evaluated CD244 in CD11b^+^Ly-6G^+^ cells. We confirmed high CD244 expression levels in CAM-treated CD11b^+^Ly6G^+^ cells (Fig 2E).

To further investigate the functions of these cell populations in vitro, bone marrow-derived macrophages (BMDMs) were co-cultured with CAM- or vehicle-treated CD11b^+^Gr-1^+^ cells stimulated with LPS (i.e., with cell–cell contact), and cytokine secretion was assessed 12 h later. Notably, CAM-treated CD11b^+^Gr-1^+^ cells suppressed pro-inflammatory cytokine production (TNF-α and IFN-γ) and increased anti-inflammatory IL-10 levels (Fig 2F–2H). Collectively, these in vitro results indicate that CAM-treated CD11b^+^Gr-1^+^ cells potentiate immunomodulatory properties related to cytokine production.

### Potency of other macrolides in CD11b^+^Gr-1^+^ cell expansion

Previous studies have suggested that 14- and 15-membered ring, but not 16-membered ring macrolides have immunomodulatory properties [19, 20]; thus, we evaluated the immunomodulatory properties of various macrolides by assessing CD11b^+^Gr-1^+^ cell expansion. Interestingly, CAM (14-membered ring) and azithromycin (15-membered ring), but not josamycin (JOS) (16-membered ring), increased the percentage and number of splenic and lung CD11b^+^Gr-1^+^ cells (S7 Fig). Because 14- and 15-, but not 16-membered macrolide antibiotics can decrease pro-inflammatory cytokine and chemokine secretion, these results suggest that the extent of macrolide-induced CD11b^+^Gr-1^+^ cell expansion is an indicator of their immunomodulatory effects.

### CAM improves survival in a mouse model of LPS-endotoxin shock via CD11b^+^Gr-1^+^cells

**Fig 3:**
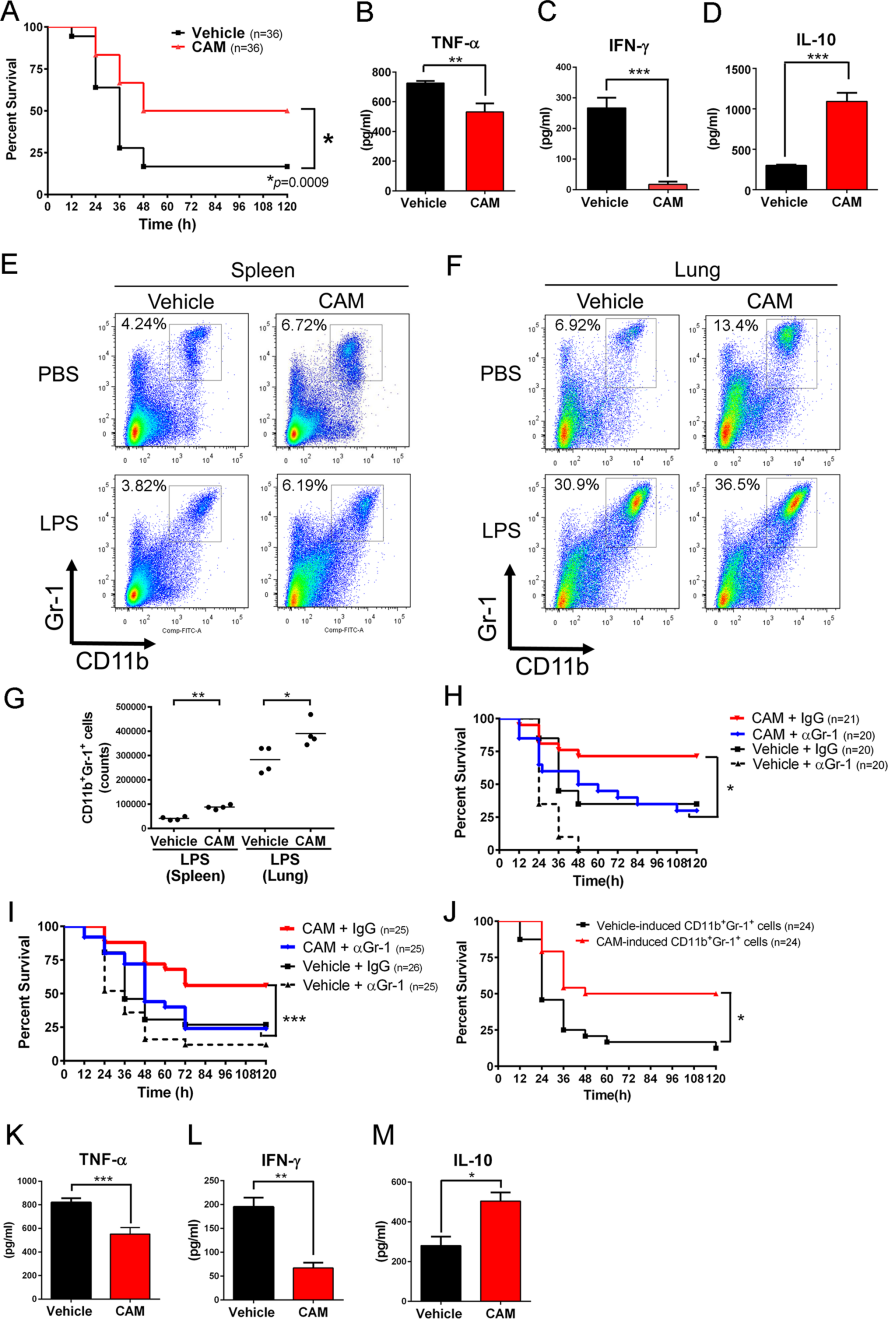
CAM ameliorates LPS-endotoxin shock via the essential contribution of CD11b^+^Gr-1^+^ cells. (A) Survival rate for LPS (50 mg/kg)-endotoxin shock in mice pretreated with vehicle or CAM (100 mg/day) daily for three consecutive days (n = 36 per group). *p = 0.0009 by the log-rank test. (B–D) Cytokine profiles in serum 12 h after LPS challenge in vehicle- or CAM-treated mice: TNF-α (B), IFN-γ (C), and IL-10 (D). (n = 5–6 per group). Data are represented as the mean ± SEM. **p < 0.01. ***p < 0.001 by the Mann–Whitney U-tests. (E and F) Representative two-parameter dot plots of CD11b^+^Gr-1^+^ cells in the spleen (E) and lungs (F) of mice intraperitoneally treated with vehicle or CAM (100 mg/day) daily for three consecutive days, followed by intraperitoneal injection with PBS or LPS (50 mg/kg) (n = 4 per group). (G) Quantification of CD11b^+^Gr-1^+^ cells in the spleen and lungs sorted from intraperitoneally vehicle- and CAM-treated (once a day for 3 days), followed by intraperitoneally LPS-treated mice (n = 4 per group). *p < 0.05, **p < 0.01 by Mann–Whitney U-tests. (H) Survival rate for LPS-endotoxin shock in vehicle- and CAM-injected mice pretreated with either anti-Gr-1 antibody (250 μg/mouse) or control IgG (n = 20–21 per group) 24 h before LPS challenge. *p = 0.0128 by the log-rank test. (I) Survival rate for LPS-endotoxin shock in vehicle- and CAM-injected mice pretreated with either anti-Gr-1 antibody (250 μg/mouse) or control IgG (n = 25–26 per group) 1 h before initiation of CAM treatment (i.e., 73 h before LPS challenge). Combined data for two independent experiments are shown. ***p < 0.001 by the log-rank test. (J) Adoptive transfer of CAM-treated CD11b^+^Gr-1^+^ cells improved the survival rate in LPS endotoxin shock (n = 24 per group). *p = 0.0023 by the log-rank test. (K-M) TNF-α (K), IFN-γ (L), and IL-10 (M) levels in serum at 12 h after intraperitoneal LPS injection (n = 5–6 per group). Data are presented as the mean ± SEM. *p < 0.05. **p < 0.01. ***p < 0.001 by the Mann–Whitney U-tests.

To examine the immunomodulatory properties of CAM-treated CD11b^+^Gr-1^+^ cells in vivo, mice were subjected to LPS-endotoxin shock. Pretreatment with CAM significantly increased survival (Fig 3A). Moreover, TNF-α and IFN-γ serum levels were significantly lower in CAM-treated mice 12 h after LPS injection, and IL-10 levels were significantly higher than those in vehicle-treated counterparts (Fig 3B–3D). A flow cytometry analysis revealed that CAM accelerated the LPS-dependent expansion of CD11b^+^Gr-1^+^ cells in both the spleen and lungs (Fig 3E and 3F). The numbers of CD11b^+^Gr-1^+^ cells in the spleen and lungs were slightly, but significantly higher in CAM-treated mice than in vehicle-treated mice (Fig 3G).

To investigate the role of the CD11b^+^Gr-1^+^ cell population in the LPS-shock model, we attempted to deplete CD11b^+^Gr-1^+^ cells. The experimental schema for the analysis summarized in Fig 3H and 3I is shown in S8 Fig. Because >85% of splenic and lung Gr-1^+^ cells in CAM-treated LPS-shock mice were CD11b^+^ (Fig 3E and 3F), we used an anti-Gr-1 (including Ly-6G and Ly-6C) antibody to deplete this cell population. The improved survival observed for CAM treatment in the LPS-shock model was reversed by Gr-1^+^ cell depletion at 24 h before LPS treatment (Fig 3H). In addition, we obtained similar results when the Gr-1^+^ cell population was depleted 1 h before the first CAM treatment (i.e., from the beginning of CAM treatment) (Fig 3I). These results indicate that the CAM-treated Gr-1 population has protective effects in this model. Next, we adoptively transferred CD11b^+^Gr-1^+^ cells isolated from CAM- or vehicle-treated donors to LPS-challenged recipient mice. Adoptive transfer of CAM-treated CD11b^+^Gr-1^+^ cells protected mice from LPS-induced lethality, as compared to vehicle-treated CD11b^+^Gr-1^+^ cells (Fig 3J) and PBS control injections (S9 Fig), and was associated with decreased TNF-α and IFN-γ and increased IL-10 serum levels (Fig 3K–3M). Collectively, these results indicated that CAM ameliorates LPS-induced lethality via CAM-treated CD11b^+^Gr-1^+^ cells.

### IL-10 production in CD11b^+^Gr-1^+^ cells protects against LPS-induced endotoxin shock

**Fig 4:**
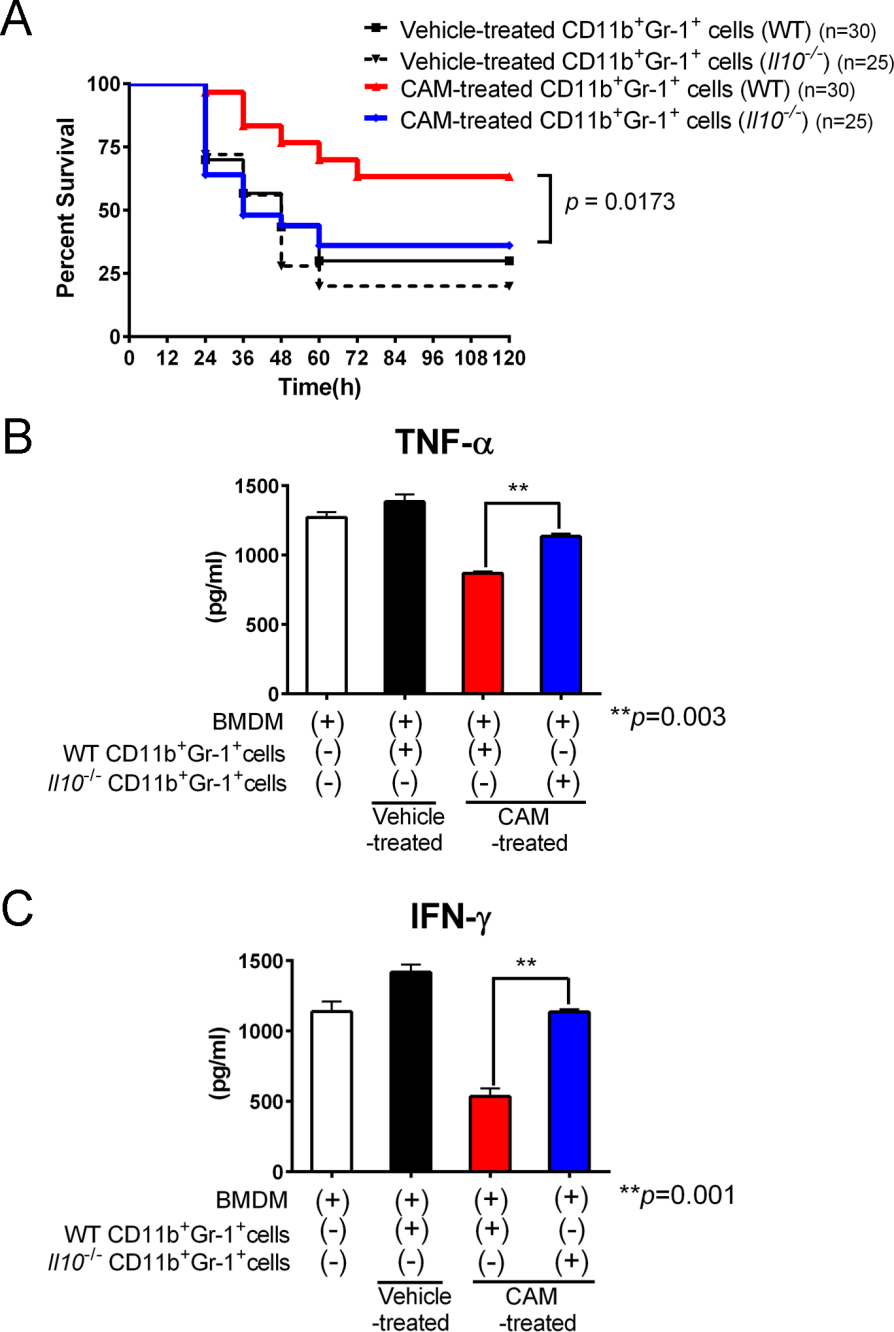
IL-10 production by CAM-treated CD11b^+^Gr-1^+^ cells is beneficial for LPS endotoxin shock. (A) CAM-treated or vehicle-treated CD11b^+^Gr-1^+^ cells in the spleen sorted from either IL-10 knockout or WT mice were adoptively transferred into LPS endotoxin-treated recipients (n = 25–30 per group). Combined data for two independent experiments are shown. *p* = 0.0137 by the log-rank test. (B and C) Levels of the pro-inflammatory cytokines TNF-α (B) and IFN-γ (C) in the supernatant of WT BMDMs co-cultured with either WT or IL-10 knockout CD11b^+^Gr-1^+^ cells in the spleen treated with vehicle or CAM (n = 3 per group). Data are presented as the mean ± SEM. **p < 0.01 by a one-way ANOVA with Tukey’s multiple comparison tests.

Because CAM-treated CD11b^+^Gr-1^+^ cells potentiated anti-inflammatory IL-10 secretion in response to LPS stimulation (Fig 2F), we hypothesized that IL-10 production in CD11b^+^Gr-1^+^ cells contributed to the beneficial effects on LPS-induced shock. To evaluate our hypothesis, we adoptively transferred CAM-treated CD11b^+^Gr-1^+^ cells from WT and IL-10 knockout (*Il10*^*-/-*^) donors into syngeneic WT recipients. Notably, WT CD11b^+^Gr-1^+^ cell grafts improved survival in the LPS-induced shock model, whereas those from *Il10*^*-/-*^ donors failed to convey these effects (Fig 4A), indicating that IL-10 production from CAM-treated CD11b^+^Gr-1^+^ cells contributed to the protective effects of macrolides in LPS-induced endotoxin shock. To confirm these effects in vitro, we measured supernatant cytokine levels in co-cultures of BMDMs and CAM-treated WT or *Il10*^-/-^ CD11b^+^Gr-1^+^ cells 12 h after LPS stimulation. As expected, the TNF-α and IFN-γ levels were significantly higher in cells co-cultured with *Il10*^-/-^ cells than in those from WT counterparts (Fig 4B and 4C), indicating that the decreased pro-inflammatory cytokine production by CAM-treated CD11b^+^Gr-1^+^ cells was at least partially IL-10-dependent.

We further investigated the role of IL-10 in expansion of the CAM-treated CD11b^+^Gr1^+^ cell population. CAM still expanded CD11b^+^Gr-1^+^ cells in *Il10*^-/-^ mice, suggesting that the major expansion mechanism of CD11b^+^Gr1^+^ cells by CAM is not related to IL-10 (S10 Fig).

### CAM improves the survival of mice with post-influenza pneumococcal pneumonia via CD11b^+^Gr-1^+^cells

**Fig 5:**
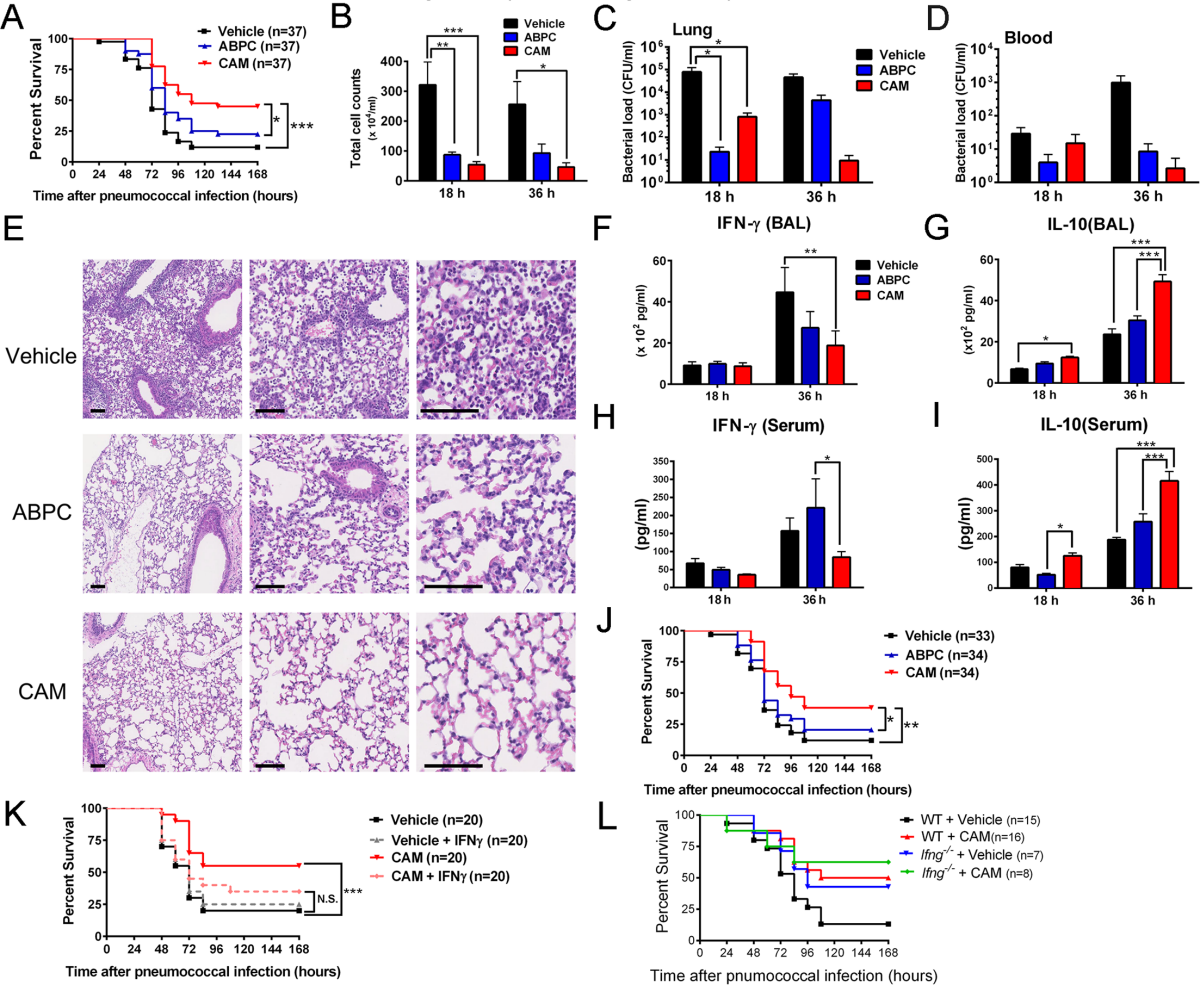
CAM improves survival in post-influenza pneumococcal pneumonia via an essential contribution of CD11b^+^Gr-1^+^ cells. (A) Survival rate of post-influenza pneumococcal pneumonia mice treated with vehicle, ampicillin (ABPC) (100 mg/kg), or clarithromycin (CAM) (100 mg/kg) (n = 37 per group). *p < 0.05, ***p < 0.001 by the log-rank test. (B) Cell counts in bronchoalveolar lavage fluid (BALF) obtained from mice with post-influenza pneumococcal pneumonia treated with vehicle, ABPC (100 mg/kg), or CAM (100 mg/kg) (n = 7–8 per group). Data are presented as the mean ± SEM. *p < 0.05. **p < 0.01, ***p < 0.001 by a two-way ANOVA with Tukey’s multiple comparison tests. (C and D) Bacterial load in the lungs (C) and blood (D) of post-influenza pneumococcal pneumonia mice treated with vehicle, ABPC (100 mg/kg), or CAM (100 mg/kg) at 18 and 36 h after pneumococcal infection (n = 15–16 per group). Data are presented as the mean ± SEM. *p < 0.05 by a two-way ANOVA with Tukey’s multiple comparison tests. (E) Lung H&E staining at 48 h after pneumococcal infection. Representative data for 5 mice per group are shown. Scale bar, 100 μm. (F–I) Levels of IFN-γ (F) and IL-10 (G) in BALF were measured by ELISA. The levels of IFN-γ (H) and IL-10 (I) in serum were measured by ELISA (n = 7–8 per group). Data are presented as the mean ± SEM. *p < 0.05. **p < 0.01. ***p < 0.001 by a two-way ANOVA with Tukey’s multiple comparison tests. (J) Survival rate of mice following adoptive transfer of CD11b^+^Gr-1^+^ cells treated with vehicle, ABPC (100 mg/kg), or CAM (100 mg/kg) in post-influenza pneumococcal pneumonia mice (n = 34 per group). Combined data for two independent experiments are shown. *p < 0.05. **p < 0.01 by the log-rank test. (K) Survival rate of vehicle-, ABPC-, and CAM-treated mice intranasally inoculated with recombinant IFN-γ (16 μg/kg) or PBS at 30 min and 24 h after pneumococcal infection (n = 20 per group). (L) Survival rate of vehicle- or CAM-treated WT and *Ifng*^*-/-*^ mice with post-influenza pneumococcal pneumonia (n = 7–16 per group).

We examined the immunomodulatory effects of CAM-treated CD11b^+^Gr-1^+^ cells in a mouse model of post-influenza pneumococcal pneumonia. In particular, we used a clinically isolated strain of *Streptococcus pneumoniae* (serotype 3) that harbors penicillin and macrolide resistance genes, although the strain was classified as a penicillin-intermediate resistant *S*. *pneumoniae* (PISP), as described in the Materials and Methods section and S1 Table. CAM treatment significantly improved survival when compared to that of vehicle- or ampicillin (ABPC)-treated mice (Fig 5A), whereas JOS treatment did not improve survival (S11 Fig). Interestingly, both CAM and ABPC significantly reduced total cell counts in bronchoalveolar lavage fluid (BALF) as compared to those in vehicle-treated mice (Fig 5B), as well as the bacterial load in lungs 18 h after infection (Fig 5C). Blood cultures showed lower bacterial loads in CAM-treated mice than in vehicle- and ABPC-treated mice, but the differences were not significant (Fig 5D). Moreover, a histopathological examination of the lungs 18 h after pneumococcal infection indicated that both CAM and ABPC ameliorated white blood cell recruitment (mainly neutrophils), alveolar wall thickening, and edema when compared to those of vehicle-treated mice (Fig 5E). Furthermore, IFN-γ levels were significantly suppressed in the BALF and serum in CAM-treated mice as compared to vehicle-treated and ABPC-treated mice, respectively, whereas IL-10 was markedly increased in both the BALF and serum of CAM-treated mice (Fig 5F–5I). Similarly, adoptive transfer of splenic CD11b^+^Gr-1^+^cells into mice with post-influenza pneumococcal pneumonia immediately after pneumococcal inoculation revealed that CAM-treated CD11b^+^Gr-1^+^ cells prolonged the survival of recipient mice when compared to ABPC- or vehicle-treated counterparts (Fig 5J).

Previous studies of post-influenza pneumococcal pneumonia have reported that IFN-γ production is detrimental to secondary bacterial infection [21, 22]; thus, because our results showed that CAM and CAM-treated CD11b^+^Gr-1^+^ cells suppress IFN-γ production in post-influenza pneumococcal pneumonia, we hypothesized that IFN-γ might have a detrimental effect on survival in our model mice. Indeed, CAM-mediated prolonged survival was partially reversed by intranasal administration of recombinant IFN-γ (Fig 5K). To further investigate the roles of IFN-γ in CAM-treated CD11b^+^Gr-1^+^ cells, IFN-γ deficient (*Ifng*^*-/-*^) and WT mice were subjected to post-influenza pneumococcal pneumonia. We first confirmed better survival in vehicle-treated *Ifng*^*-/-*^ mice than in vehicle-treated WT mice (Fig 5L), consistent with previous results showing that *Ifng*^*-/-*^ mice are resistant to post-influenza pneumococcal pneumonia [22]. We also observed slightly higher survival in CAM-treated *Ifng*^*-/-*^ mice than in vehicle-treated *Ifng*^*-/-*^ mice and CAM-treated WT mice (Fig 5L), suggesting that the protective effects of CAM in this model depend, at least in part, on suppression of IFN-γ production. Collectively, these data demonstrate that CAM improves the overall survival of mice with post-influenza pneumococcal pneumonia, at least in part via suppression of IFN-γ production by CAM-treated CD11b^+^Gr-1^+^ cells.

Previous studies have indicated that type I IFN is also detrimental in a post-influenza pneumococcal pneumonia model using interferon-α/β receptor (IFNAR) knockout (*Ifnar1*^*-/-*^) mice [23]. To investigate the role of type I IFN in CAM-treated CD11b^+^Gr-1^+^ cells, *Ifnar1*^*-/-*^ mice and WT mice were subjected to post-influenza pneumococcal pneumonia. We did not observe a significant difference between WT mice and *Ifnar1*^*-/-*^ mice. In addition, CAM did not significantly improve survival in *Ifnar1*^*-/-*^ mice, but it resulted in a slight improvement in survival in *Ifnar1*^*-/-*^ mice (S12 Fig). These results may be explained by the strong virulence of influenza in *Ifnar1*^*-/-*^ mice. Accordingly, we could not determine whether the protective effects of CAM in post-influenza pneumococcal pneumonia are independent or partially dependent on type I IFN, and additional studies with larger sample sizes are needed.

### CAM expanded CD11b^+^Gr-1^+^ cells via the STAT3-Bv8 axis

We next explored the mechanism underlying CAM-induced CD11b^+^Gr-1^+^ cell expansion, and initially focused on effectors known to induce the differentiation and proliferation of CD11b^+^Gr-1^+^ cells, including IL-17, CCL2, CXCR4, and toll-like receptor (TLR)/MyD88 signaling [10]. Thus, we examined CAM-mediated CD11b^+^Gr-1^+^ cell expansion in *Il17a*^-/-^, *Ccl2*^-/-^, and *Myd88*^-/-^ mice, as well as WT mice treated with a CXCR4 antagonist (AMD3100). However, no differences were observed between these mice and WT controls (S13 Fig), indicating that IL-17, CCL2, CXCR4, and TLR/MyD88 signaling pathways are not involved in this process.

**Fig 6:**
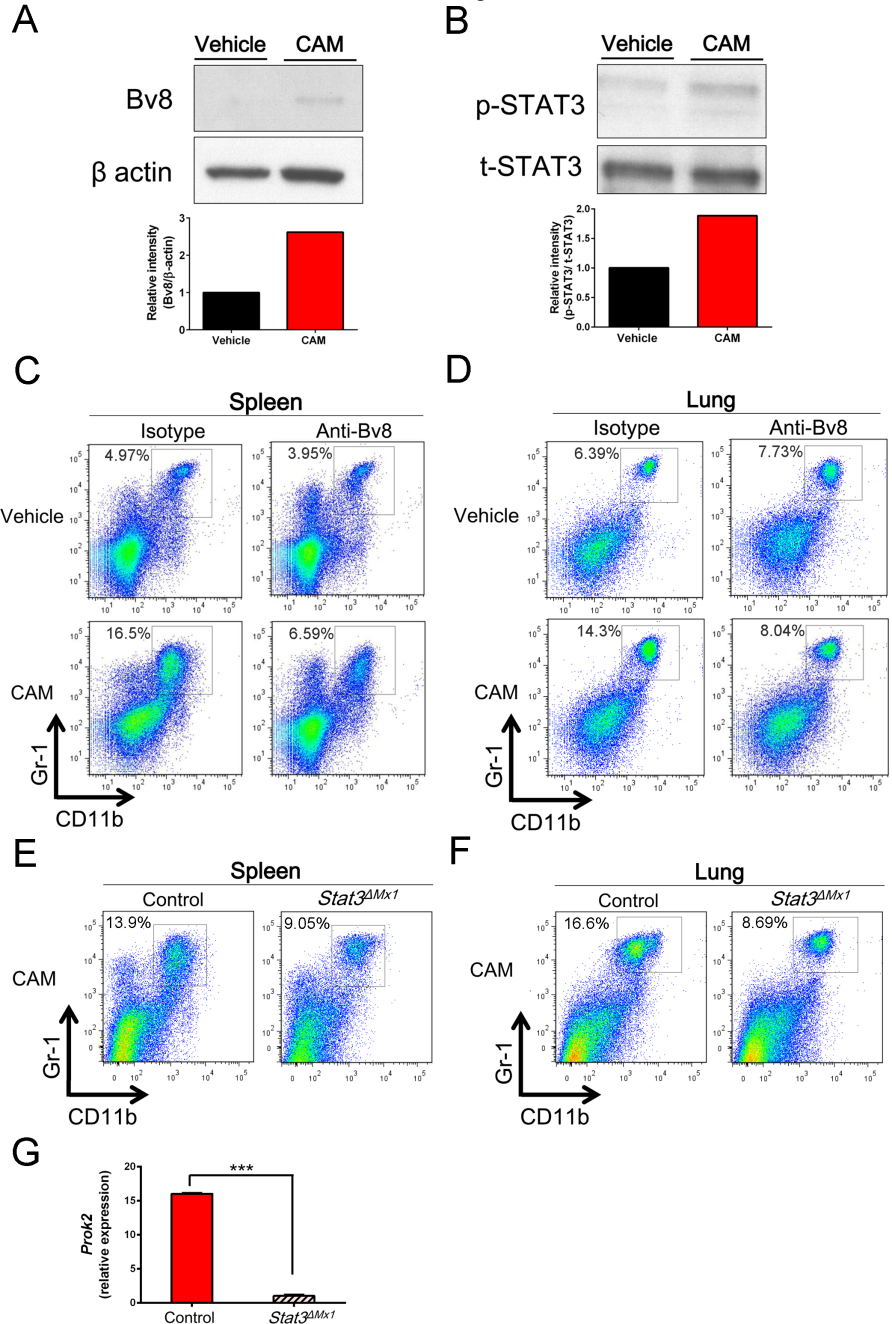
CAM expands the CD11b^+^Gr-1^+^ cell population via the STAT3-Bv8 axis. (A and B) Representative immunoblotting of Bv8 (A) and phosphorylated (p)-STAT3 (B). Beta-actin and total (t) STAT3 were used as loading controls. Relative band intensities ware quantified by densitometry. (C and D) Representative two-parameter dot plots of CD11b^+^Gr-1^+^ cells in the spleen (C) and lungs (D) sorted from mice intraperitoneally treated with vehicle or CAM (100 mg/day) daily for three consecutive days. Mice were intraperitoneally injected with an anti-Bv8 antibody (5 mg/kg) or control IgG at 4 days and 1 day before CAM treatment and daily for three consecutive days during CAM treatment (n = 4 per group). (E and F) Representative two-parameter dot plots of CD11b^+^Gr-1^+^ cells in the spleen (E) and lungs (F) sorted from poly(I:C)-treated *Stat3*^*flox/flox*^/Mx1-Cre (*Stat3*^Δ*Mx1*^) mice and control *Stat3*^*flox/flox*^ mice that were intraperitoneally treated with vehicle or CAM (100 mg/day) daily for three consecutive days (n = 4 per group). (G) Bv8 (*Prok2*) mRNA expression in CD11b^+^Gr-1^+^ cells sorted from *Stat3*^Δ*Mx1*^ mice and control *Stat3*^*flox/flox*^ mice (n = 4 per group). Data are presented as the mean ± SEM. ***p < 0.001 by the Mann–Whitney U-test.

The prokineticin family member Bv8 (also known as prokineticin 2 [Prok2]) was previously reported to induce CD11b^+^Gr-1^+^ cells in an autocrine and paracrine STAT3-dependent manner [24]. Notably, our microarray data identified that Bv8 (*Prok2*) was highly expressed (12.3-fold higher relative to WT mice) in splenic CD11b^+^Gr-1^+^ cells obtained from CAM-treated mice (Fig 2A). A protein analysis of splenic cells after CAM treatment revealed higher Bv8 expression than that in vehicle-treated mice (Fig 6A), as well as increased phosphorylated (p)-STAT3 (Fig 6B). To investigate the role of Bv8 in CAM-induced CD11b^+^Gr-1^+^ cell induction, mice were administered an anti-Bv8 antibody at 4 days and 1 day before, and then during, the CAM treatment period (three consecutive days). Notably, treatment with the anti-Bv8 antibody reduced the percentage of CD11b^+^Gr-1^+^ cells in both the spleen (16.5% vs. 6.59%; 60.1% reduction) (Fig 6C) and lungs (14.3% vs. 8.04%; 43.8% reduction) (Fig 6D). Because Bv8 and STAT3 form a feed-forward loop that promotes CD11b^+^Gr-1^+^ myeloid cell proliferation [24], we investigated this as a potential induction mechanism in *Stat3*^*flox/flox*^*/Mx1-Cre* (*Stat3*^Δ*Mx1*^) mice. As predicted, the CAM-treated CD11b^+^Gr-1^+^ cell populations in the spleen and lungs were reduced in *Stat3*^Δ*Mx1*^ mice (Fig 6E and 6F). Consistently, *Bv8* gene expression was significantly reduced in splenic CD11b^+^Gr-1^+^ cells isolated from CAM-treated *Stat3*^Δ*Mx1*^ mice when compared to expression in *Stat3*^*flox/flox*^ control mice (Fig 6G). Taken together, these results indicate that STAT3-Bv8 signaling facilitates the CAM-mediated expansion of the CD11b^+^Gr-1^+^ cell population.

### Gut commensal microbiota may contribute to CAM-induced CD11b^+^Gr-1^+^ cell expansion

Macrolide administration causes substantial changes in the composition of the gut microbiota [25]. Accordingly, we hypothesized that the modulation of the gut microbiota by CAM might contribute to the expansion of CD11b^+^Gr-1^+^ cells. To investigate this hypothesis, germ-free mice and specific pathogen-free (SPF) mice were administered CAM daily for three consecutive days, and the CD11b^+^Gr-1^+^ cell populations were quantified the day after the last injection. The baseline CD11b^+^Gr-1^+^ cell population in untreated germ-free mice was significantly lower than that in untreated SPF mice (S14 Fig), suggesting that the resident CD11b^+^Gr-1^+^ cell population is influenced by the gut commensal microbiota. Moreover, expansion of the CD11b^+^Gr-1^+^ cell population by CAM was also observed in both germ-free mice and SPF mice; however, the relative expansion of splenic and lung CD11b^+^Gr-1^+^ cells was significantly lower in CAM-treated germ-free mice than in SPF counterparts, suggesting that commensal gut microbiota enhance CD11b^+^Gr-1^+^ cell population expansion in response to CAM treatment (S14 Fig).

### CAM modulates the immunosuppressive properties of Lineage^−^HLA-DR^−^CD11b^+^CD33^+^ cells in humans

**Fig 7:**
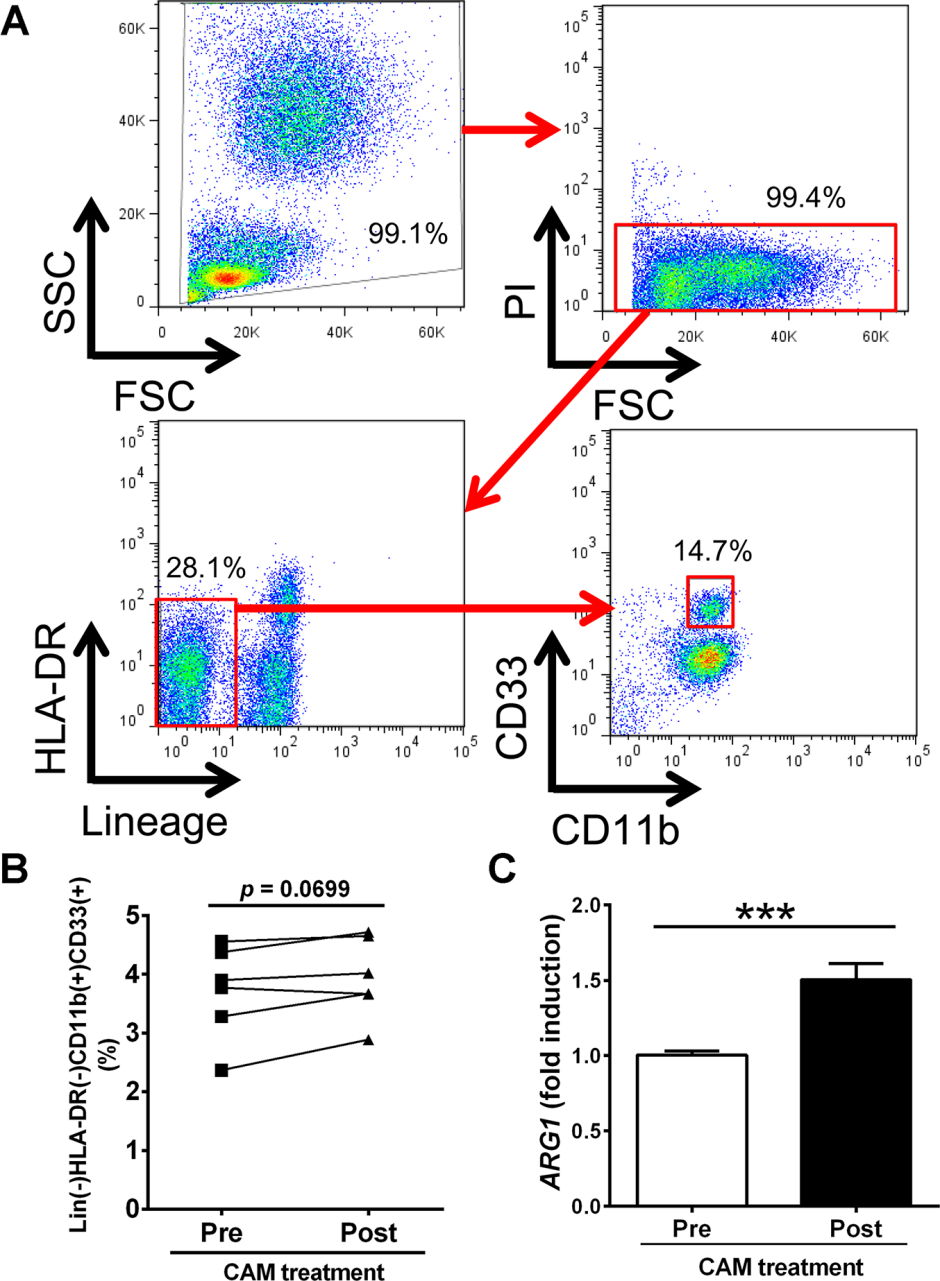
Immunosuppressive properties of human Lineage^-^HLA-DR^-^CD11b^+^CD33^+^ cells expanded by CAM treatment. (A) Representative two-parameter dot plots of Lineage^-^HLA-DR^-^CD11b^+^CD33^+^ cells of healthy volunteers evaluated by flow cytometry (n = 6). PI, propidium iodide. (B) Percentage of Lineage^-^HLA-DR^-^CD11b^+^CD33^+^ cells before and 7 days after oral treatment with CAM (800 mg/day). Data were analyzed by the Wilcoxon matched-pairs signed rank test. (C) Expression of *ARG1* in sorted Lineage^-^HLA-DR^-^CD11b^+^CD33^+^ cells from healthy volunteers before and 7 days after oral treatment with clarithromycin (CAM) (800 mg/day) (n = 6). Data are presented as the mean ± SEM. ***p < 0.001 by the Mann–Whitney U-test.

To determine whether the immunosuppressive MDSC-like population also exhibits CAM-induced expansion in humans, six healthy volunteers were administered 800 mg of CAM daily for 7 days and relative changes in the Lineage^*−*^HLA-DR^*−*^CD11b^+^CD33^+^ MDSC population were examined in peripheral blood by flow cytometry (Fig 7A). Although the Lineage^*−*^HLA-DR^*−*^CD11b^+^CD33^+^ cell population appeared to increase following CAM treatment, the difference was not significant (*p =* 0.0699) (Fig 7B); however, arginase-1 gene expression was markedly elevated in these cells after 7 days of CAM treatment compared to the baseline control measurements (Fig 7C). These results suggest that human Lineage^−^HLA-DR^−^CD11b^+^CD33^+^ MDSCs were polarized to a more immunosuppressive phenotype in response to CAM treatment.

## Discussion

In this study, we demonstrated that CAM stimulates the expansion of the immunosuppressive CD11b^+^Gr-1^+^ cell population to enhance survival in a LPS-induced shock and post-influenza pneumococcal pneumonia mouse model, in a STAT3/Bv8 axis-dependent manner. We also showed that human Lineage^*−*^HLA-DR^*−*^CD11b^+^CD33^+^ cells correspond to the CD11b^+^Gr-1^+^ population in mice, as they both exhibited a significant increase in arginase-1 mRNA expression levels following CAM treatment.

Increasing evidence suggests that macrolides have immunomodulatory properties, beyond their antibacterial effects [1, 2]; however, the overuse of these drugs for this purpose is criticized because the mechanism of action is unclear [26]. Therefore, the elucidation of their exact immunomodulatory effects is needed. Numerous in vitro studies have demonstrated that macrolides modulate the release of humoral factors from immune, epithelial, and other structural cells [8]. Our study is distinguished from previous reports in that it identified the unique immunosuppressive cell population mediating these effects in mice, and implicates a homologous cell population in humans.

Our analyses indicated that the CAM-treated CD11b^+^Gr-1^+^ cells were MDSC-like cells. MDSCs are a population comprised of heterogeneous cell clusters consisting of myeloid progenitor cells and immature myeloid cells, e.g., immature neutrophils [10]. The differences between granulocytic-MDSCs and mature neutrophils are often debatable [27, 28]. Here, we confirmed that the CAM-treated CD11b^+^Gr-1^+^ cell population includes both CD11b^+^Ly6G^+^Ly6C^low^ cells (granulocytic-MDSC-like cells) and CD11b^+^Ly-6G^*−*^Ly-6C^+^ cells (monocytic-MDSC-like cells). We cannot estimate the magnitude of the contributions of these two populations (granulocytic- and monocytic-MDSC-like cells) to the beneficial effects of CAM treatment because we used an anti-Gr-1 antibody (RB6-8C5 clone, anti-Ly-6G and Ly-6C antibody) to deplete the Gr-1^+^ cell population. The CAM-treated CD11b^+^Gr-1^+^ cell population can be discriminated from mature neutrophils in their anti-inflammatory effects, as mature neutrophils are generally inflammatory in nature. We showed that CD11b^+^Gr-1^+^ cells actively secrete the anti-inflammatory cytokine IL-10 and suppress the production of the inflammatory cytokines IFN-γ and TNF-α, although selected neutrophils have also been reported to produce IL-10 [29]. In addition, our results indicating that elastase activity and MPO activity were lower in CAM-treated CD11b^+^Gr-1^+^ cells than in activated neutrophils/monocytes/macrophages, including LPS-treated CD11b^+^Gr-1^+^ cells and thioglycolate-induced neutrophils, are consistent with the notion that CAM-treated CD11b^+^Gr-1^+^ cells are not activated neutrophils, but MDSC-like cells.

Importantly, our work demonstrated that CAM-treated CD11b^+^Gr-1^+^ cells potentiate the production of IL-10, an anti-inflammatory cytokine that plays a central role in infection by limiting the immune response to pathogens and preventing damage to the host [30]. Many earlier studies found that IL-10 promotes survival in sepsis and endotoxin shock [31–33], consistent with our results indicating beneficial effects of IL-10 in LPS-induced shock. IL-10 has been reported to have detrimental roles in post-influenza pneumococcal pneumonia [34, 35]; however, another previous study found no significant differences in susceptibility to post-influenza secondary pneumococcal infection between WT and *Il10*^*-/-*^ mice [22]. We did not determine the detailed mechanisms underlying increased IL-10 levels in the post-influenza pneumococcal pneumonia model in the present study, but this should be evaluated in further studies.

We also showed that CAM-treated CD11b^+^Gr-1^+^ cells distinctly suppressed IFN-γ production both in vitro and in vivo, corroborating earlier studies of the 14-membered ring macrolide erythromycin in a mouse model of influenza virus infection [36]. The significance of suppressed IFN-γ production in LPS-induced endotoxin shock was not evaluated in the present study; however, IFN-γ has been reported to be detrimental in LPS-endotoxin shock by hampering endotoxin tolerance [37, 38]. Considering these previous reports and our results indicating the partial restoration of IFN-γ production in macrophages co-cultured with CAM-treated *Il10*^*-/-*^ CD11b^+^Gr-1^+^ cells (Fig 4C), we speculate that enhanced IL-10-mediated IFN-γ suppression by CAM would also protect against LPS-induced endotoxin shock. In terms of significance, a previous study indicated that IFN-γ production by CD4^+^ and CD8^+^ T cells in response to influenza virus infection enhanced susceptibility to secondary pneumococcal infection [22]. In addition, accumulating evidence indicates that CD11b^+^Gr-1^+^ cells suppress IFN-γ production by T cells [39, 40] and NK cells [41]. As such, we speculate that the suppression of IFN-γ production in these cells, as well as macrophages, by CAM treatment would have protective effects in post-influenza pneumococcal pneumonia. Consistent with this notion, we confirmed that exogenous administration of recombinant IFN-γ partially reversed the survival benefit in post-influenza pneumococcal pneumonia model mice. We also observed slightly greater survival in CAM-treated *Ifng*^-/-^ mice than in vehicle-treated *Ifng*^-/-^ mice and CAM-treated WT mice. These results suggest that the protective effects of CAM in this model at least partially depend on suppression of IFN-γ production. We speculate that, in addition to IFN-γ suppression, other mechanisms, such as increased IL-10, contribute to the protective effects of CAM in this model.

Furthermore, we found that the STAT3/Bv8 axis is actively involved in the mechanism underlying CAM-induced CD11b^+^Gr-1^+^ cell expansion. Bv8 induces CD11b^+^Gr-1^+^ cells in an autocrine and paracrine STAT3-dependent manner [24], and regulates CD11b^+^Gr-1^+^ cell-dependent tumor angiogenesis [42]. A Bv8 antagonist was found to inhibit angiogenesis and myeloid cell infiltration in mouse models of glioblastoma and pancreatic cancer [43]. These studies, in combination with our study, suggest that the STAT3/Bv8 axis is essential to myeloid cell recruitment, including that of CD11b^+^Gr-1^+^ cells. In addition, because STAT3 plays a crucial role in the IL-10-mediated anti-inflammatory response [44], and considering our results for IL-10 induction from CAM-treated CD11b^+^Gr-1^+^ cells, it is likely that the expanded CD11b^+^Gr-1^+^ cell population mediates its immunomodulatory effects via STAT3-dependent IL-10 production.

Previous studies have clearly demonstrated that 14- and 15-membered, but not 16-membered macrolide antibiotics, can decrease pro-inflammatory cytokine and chemokine secretion [8, 19, 20]. Similarly, we observed expansion of CD11b^+^Gr-1^+^ cells following treatment with 14- and 15-membered, but not 16-membered macrolide antibiotics, suggesting that the degree of CD11b^+^Gr-1^+^ cell expansion is correlated with their immunomodulatory properties. Thus, macrolide-treated CD11b^+^Gr-1^+^ cells might be a good biological marker for evaluating the potency of their immunomodulatory properties.

Oral administration of 25–100 mg/kg CAM in mice is comparable to clinically achievable levels in humans [45]. As the area under the curve (AUC) was more than 2-fold higher for intraperitoneal administration of 10 mg/kg CAM than for oral administration of 10 mg/kg CAM (personal communication), we believe that the intraperitoneal administration of CAM at 100 mg/kg in the present study is clinically relevant for humans.

The overuse of macrolide antibiotics could result in the evolution of macrolide-resistant organisms. To address this concern, immunomodulatory macrolides lacking antimicrobial effects, e.g., EM900 and EM703, have been in development [46, 47]. The presence of immunosuppressive CD11b^+^Gr-1^+^ cells can be a good surrogate marker to measure the immunomodulatory effects of these types of drugs in early development.

Finally, we tried to identify the human immunosuppressive MDSC population that corresponds to murine CD11b^+^Gr-1^+^ cells. Exact human MDSC surface markers are yet to be identified, partly owing to the extensive heterogeneity in the human MDSC population [11]. However, based on previous studies [11], we identified Lineage^-^HLA-DR^-^CD11b^+^CD33^+^ cells as a major human MDSC population. In addition, we observed upregulation of arginase-1 mRNA in the human MDSC population after oral administration of CAM, indicating that CAM likely expands immunosuppressive MDSC-like cells in humans. In the present study, peripheral blood was used to assess CAM-treated human MDSCs, but these effects could be evaluated with much greater clarity in lung or spleen cell populations, as in the model mice.

In the present study, we used an anti-Gr-1 antibody (RB6-8C5 clone, anti-Ly-6G and Ly-6C antibody) to deplete the Gr-1^+^ cell population, resulting in depletion of both monocytic- and granulocytic-MDSC-like cell populations. This may not be a good approach for the depletion of MDSC-like cells owing to the lack of selectivity, i.e., myeloid cells such as neutrophils and monocytes/macrophages can also be depleted by treatment with the anti-Gr-1 antibody. Although several parameters have been suggested for distinguishing granulocytic-MDSCs from neutrophils [14], they are insufficient [48]. Further investigations are needed to better differentiate these cells. A working hypothesis for the mechanism underlying CAM-mediated expansion of the CD11b^+^Gr-1^+^ cell population is summarized in S15 Fig. In this system, CAM expands the CD11b^+^Gr-1^+^ cell population in a STAT3/Bv8-dependent manner. The anti-inflammatory effects of these cells, including increased IL-10 and decreased IFN-γ, are sufficient to protect mice from death by LPS-induced shock and post-influenza pneumococcal pneumonia.

In conclusion, we identified a novel mechanism underlying CAM-mediated expansion of the immunosuppressive CD11b^+^Gr-1^+^ cell population, sufficient to attenuate LPS-induced lethal shock and post-influenza pneumococcal infection. Considering that the immunosuppressive effects of CD11b^+^Gr-1^+^ cells may also ameliorate various inflammatory diseases, our findings provide a new strategy for the treatment of inflammatory diseases and the development of new classes of macrolides and novel drugs. Further detailed studies would contribute to a complete understanding of the mechanism underlying the beneficial effects of CAM.

## Materials and methods

### Mice

Male WT C57BL/6J mice (8–10 weeks old) were purchased from CLEA Japan, Inc. (Tokyo, Japan). *Il10*^*−/−*^ mice and *Ccl2*^*-/-*^ *mice* were purchased from Jackson Laboratory (Bar Harbor, ME, USA). *Il17a*^*-/-*^ *and Ifng*^*-/-*^ mice were kindly provided by Prof. Yoichiro Iwakura (Tokyo University of Science, Noda, Japan). *Ifnar1*^*-/-*^ mice were kindly provided by Prof. Shigekazu Nagata (Kyoto University, Kyoto, Japan) [48, 49].

*Stat3*^*flox/flox*^ mice and *Myd88*^*-/-*^ mice were purchased from Oriental BioService, Inc. (Kyoto, Japan) Mx-1-Cre mice were kindly provided by the Mouse Genetics Cologne Foundation in Germany. *Stat3*^Δ*Mx1*^ mice were generated by mating *Stat3*^*flox/flox*^ mice with Mx-1-Cre mice. To remove the floxed *Stat3* allele, 8-week-old *Stat3*^*ΔMx1*^ mice were intraperitoneally injected with 250 μg of polyinosinic-polycytidylic acid ([Poly(I:C)]; Sigma-Aldrich, St. Louis, MO, USA) three times every other day. Littermate *Stat3*^*flox/flox*^ mice served as controls. Germ-free mice were purchased from CLEA Japan, Inc., and were kept in the germ-free facility at Keio University School of Medicine. Other mice were housed under specific pathogen-free (SPF) conditions.

### Ethics statement

The present study was performed in strict accordance with the recommendations of the Guide for the Care and Use of Laboratory Animals of the National Institutes of Health. All animal experiments were approved by the Animal Care and Use Committee at the Keio University School of Medicine (Protocol No. 12110). The human study was approved by the Ethics Committee of Keio Center for Clinical Research in November 2012 (Protocol No. 20120250). All participants of the study (i.e., blood donors) were informed about the risks of participation in the study and written consent was obtained from all of them. All participants were adults and non-minors.

### Preparation and injection of clarithromycin and other antibiotics

Clarithromycin (CAM) was kindly provided by Taisho Toyama Pharmaceutical Co., Ltd. (Tokyo, Japan), and diluted with PBS containing 5% gum arabic. The CAM or vehicle solution (5% gum arabic in PBS) was intraperitoneally injected at 100 mg/kg daily throughout the course of the experiment. In oral administration experiments, the CAM and vehicle solutions were administered at 100 mg/kg by gavage daily for 14 days. The dose of CAM was determined based on preliminary experiments summarized in S1 Fig and previous reports [45, 50, 51]. Azithromycin (Sigma-Aldrich), josamycin (Sigma-Aldrich), and ampicillin (Sigma-Aldrich) were dissolved in dimethyl sulfoxide (DMSO; Sigma-Aldrich) at 10 mg/mL and further diluted in sterile PBS. Antibiotics were administered in vivo at 100 mg/kg, 200 mg/kg, and 100 mg/kg, based on previous reports [51–54].

### Flow cytometry and sorting

In mouse studies, single-cell suspensions were prepared for flow cytometry as follows; spleens and lungs were cut into small fragments and digested for 45 min at 37°C with RPMI 1640 containing 0.05 mg/mL Liberase (Roche Diagnostics, Basel, Switzerland) and 250 U/mL DNAse I (Worthington Biochemical Corporation, Lakewood, NJ, USA). Single cells were washed with fluorescence activated cell sorting (FACS) buffer (2% fetal calf serum [FCS] and 1 mg/mL sodium azide in PBS) and incubated with an anti-CD16/CD32 monoclonal antibody (eBioscience, San Diego, CA, USA) for 10 min followed by staining with the following antibody conjugates for 20 min: PE-Cy7-CD45 (30-F11; BioLegend, San Diego, CA, USA), APC-Cy7-CD45 (30-F11; BD Biosciences, Franklin Lakes, NJ, USA), PerCP-Cy5.5-CD45 (30-F11; BD Biosciences), PE-Cy7-CD3 (17A2; BioLegend), PerCP-Cy5.5-CD4 (GK1.5; BioLegend), APC-Cy7-CD8a (53–6.7; BioLegend), APC-TCR-γδT (B1; BioLegend), PE-NK1.1 (PK136; BioLegend), APC-Gr-1 (RB6-8C5; BioLegend), FITC-CD11b (M1/70; BioLegend), APC/Cy7-Ly-6G (1A8; BioLegend), PerCP/Cy5.5-Ly-6C (HK1.4; BioLegend), PE-CD244.2 (2B4 B6; eBioscience), PE-CTLA-4 (BioLegend), PE-PD1 (eBioscience), PE-PDL1 (eBioscience), PE-CXCR2 (BioLegend), PE-CXCR4 (eBioscience), PE-CD80 (BioLegend), PE-CD115 (eBioscience), and PE-CX3CR1 (R&D Systems, Minneapolis, MN, USA). The cells were then washed twice and subsequently stained with PE-Texas Red propidium iodide (PI; BD Biosciences).

The antibody conjugates PE-CD33 (HIM3-4; BD Biosciences), Alexa700-CD11b (CR3A; BioLegend), PE/Cy7-HLA-DR (BioLegend), FITC-Lineage (CD3, CD19, CD56) (eBioscience), and APC/Cy7-CD45 (HI30; BioLegend) were used for human cell analyses. The cells were washed twice and stained with PE-Texas Red PI (BD Biosciences).

Stained cells were processed by flow cytometry using a BD FACS Aria II system (BD Biosciences) or MoFlo XDP (Beckman Coulter, Brea, CA, USA) and the data were analyzed using FlowJo 7.6.2 (Tree Star, Inc., Ashland, OR, USA).

### Immunofluorescence

Lung tissue was fixed with 4% paraformaldehyde and embedded in paraffin. The lung sections were deparaffinized in xylene and rehydrated in a graded ethanol series, and antigen retrieval was performed by heating sodium citrate buffer at 95°C for 20 min. Non-serum protein block (Dako) was applied for 30 min. Slides were then stained with rat anti-Gr-1 primary antibody (RB6-8C5; R&D Systems) or rabbit anti-arginase-1 primary antibody (ABS535; Merck, Kenilworth, NJ, USA) followed by Alexa Fluor 488- or Alexa Fluor 555-coupled secondary antibody, respectively (Invitrogen, Carlsbad, CA, USA). Sections were counterstained with DAPI (Vector Labs) and analyzed using an Axio Imager (Zeiss, Oberkochen, Germany).

### In vivo antibody labeling

After mice were anesthetized by intraperitoneal injection of xylazine (10 mg/kg) and ketamine (100 mg/kg), an APC-Cy7-CD45 antibody conjugate (0.4 μg/2 μL diluted in 100 μL of PBS) was administered 5 min before euthanasia together with heparin (10 IU/g). After 5 min, mice were exsanguinated and intratracheally injected with a PerCPCy5.5-CD45 antibody conjugate (0.4 μg/2 μL diluted in 100 μL of PBS). After an additional 5 min, the lungs were harvested and a lung single cell suspension was prepared, followed by staining with a PE-Cy7-CD45 antibody conjugate ex vivo.

### Microarray analysis

Total RNA was isolated using the RNeasy Mini Kit (QIAGEN, Hilden, Germany) according to the manufacturer’s protocol. The isolated RNA was quantified, and quality was assessed using an Agilent 2100 BioAnalyzer with the RNA Nano Chip (Agilent, Santa Clara, CA, USA). Then, 2 μg of total RNA was used to generate single-stranded antisense cDNA using the Ovation Biotin System (NuGEN Technologies, San Carlos, CA, USA) according to the manufacturer’s instructions. Labeled targets were hybridized to Affymetrix MOE430 2.0 GeneChip microarrays (Affymetrix) for 16 h at 45°C. The arrays were washed and scanned according to standard Affymetrix protocols. GeneSpring 12.0 (Silicon Genetics, Redwood City, CA, USA) was used for data analysis. Microarray data sets have been deposited in the National Center for Biotechnology Information (NCBI) Gene Expression Omnibus under accession number GSE81241.

### Arginase activity

Splenic CD11b^+^Gr-1^+^ cells sorted from CAM- or vehicle-treated mice were lysed in 50 μL of RIPA Lysis and Extraction Buffer (Thermo Fisher Scientific, Waltham, MA, USA) supplemented with Halt Protease Inhibitor Cocktail and Phosphatase Inhibitor Cocktail 2 (Sigma-Aldrich). The lysate was collected by centrifugation at 14,000 × *g* at 4°C for 15 min and 100 μL of ddH_2_O was added. Arginase activity was determined using the QuantiChrom Arginase Assay Kit (BioAssay Systems, Hayward, CA, USA) following the manufacturer's instructions.

### Nitric oxide measurement

The NO concentration in spleen extracts was determined using the Quantichrom Nitric Oxide Assay Kit (BioAssay Systems) according to the manufacturer’s instructions.

### T cell proliferation assay

CD3^+^ T cells were isolated from splenocytes by negative selection, using a magnetic cell sorting system (MACS; Miltenyi Biotec, Bergisch Gladbach, Germany). Sorted cells were washed and resuspended in 5 mL of PBS/0.1% bovine serum albumin (BSA). T cell proliferation assays were performed using the CellTrace CFSE Cell Proliferation Kit (Thermo Fisher Scientific) according to the manufacturer’s instructions. Briefly, the sorted CD3^+^ T cells were incubated with CFSE for 20 min, washed, and then stimulated with anti-CD3 antibody (5 μg/mL) (BioLegend) and anti-CD28 antibody (1 μg/mL) (BioLegend). The CFSE-labeled CD3^+^ T cells were then co-cultured with sorted splenic CAM-treated or vehicle-treated CD11b^+^Gr-1^+^ cells for 3 days prior to fluorescence intensity analysis by flow cytometry.

### Elastase activity assay

The Neutrophil Elastase Activity Assay Kit (Cayman, Ann Arbor, MI, USA) was used to measure elastase activity according to the manufacturer’s instructions.

### Myeloperoxidase activity assay

The Myeloperoxidase (MPO) Activity Assay Kit (Colorimetric) (ab105136; Abcam, Cambridge, UK) was used to measure MPO activity according to the manufacturer’s instructions.

### Phagocytic activity assay

The Phagocytosis Activity Assay Kit (IgG FITC) (Cayman) was used to measure phagocytic activity according to the manufacturer’s instructions.

### Preparation of various types of CD11b^+^Gr-1^+^ cells, neutrophils, and monocytes

To prepare LPS-treated CD11b^+^Gr-1^+^ cells, mice were intraperitonially injected with LPS (50 mg/kg), and splenic CD11b^+^Gr-1^+^ cells 12 h after LPS treatment were sorted using anti-Gr-1 magnetic beads. To prepare thioglycolate-induced neutrophils, mice were intraperitoneally injected with 3% thioglycolate in 2 mL of PBS. Peritoneal neutrophils were recovered 4 h after the injection. For isolation of peripheral neutrophils, the Cell-based Assay Neutrophil Isolation Histopaque kit was used according to the manufacturer’s instructions (Cayman). To prepare monocytes, Monocyte Isolation Kit (BM) was used according to the manufacturer’s instructions (Miltenyi Biotec).

### Culture of murine bone marrow-derived macrophages

Bone marrow cells were harvested from 8–10-week-old-male C57BL/6J mice by flushing the femur and tibia with RPMI 1640 medium. Recovered cells were then cultured in bone marrow cell medium (20% FCS, 30% L-cell supernatant, 2 mM l-glutamine, 1% penicillin/streptomycin, 0.25 μg/mL amphotericin B in RPMI 1640). Fresh bone marrow cell medium was added on day 3. On day 6, adherent cells were replated in RPMI 1640 medium supplemented with 10% FCS, 2 mM l-glutamine, and 1% penicillin/streptomycin for use as BMDMs. On day 7, the medium was removed and BMDMs were cultured with or without CD11b^+^Gr-1^+^ cells (either CAM-treated or vehicle-treated) for 1 h, followed by stimulation with LPS for 12 h.

### Enzyme-linked immunosorbent assay

TNF-α, IFN-γ, and IL-10 levels were measured using a DuoSet ELISA Kit (R&D Systems) according to the manufacturer’s instructions.

### Lipopolysaccharide-induced endotoxin shock mice model

Mice received a single intraperitoneal (i.p.) injection of 50 mg/kg LPS (*Escherichia coli* O111:B5; Sigma-Aldrich). For CAM pre-treatment, mice were intraperitoneally injected with CAM (100 mg/kg) once daily for three consecutive days, followed by LPS challenge the day after the last injection of CAM.

For the pharmacological depletion of Gr-1^+^ cells, mice received an i.p. injection of an anti-Gr-1 antibody (250 μg/mouse) (clone RB6-8C5; BioLegend) or isotype control antibody 24 h before LPS challenge or 1 h before starting CAM treatment (i.e., 73 h before LPS challenge). The antibody dose was determined according to our previous study [55].

For adoptive transfer experiments, splenic CD11b^+^Gr-1^+^ cells (1 × 10^6^/mouse) in 0.1 mL of PBS sorted from CAM- or vehicle-treated mice were intravenously transferred into mice by tail vein injection.

### Influenza virus and secondary *Streptococcus pneumoniae* infection in mice

Influenza virus A/H1N1/PR8/34 (PR8) was kindly provided by Dr. Hideki Hasegawa (National Institute of Infectious Diseases, Tokyo, Japan) [56]. The PR8 virus titer was measured by a plaque forming assay using Madin-Darby canine kidney (MDCK) cells (CCL-34; American Type Culture Collection).

*S*. *pneumoniae* was kindly provided by Dr. Kimiko Ubukata (Keio University, Tokyo, Japan). The strain was clinically isolated (serotype 3) and was both penicillin- and macrolide-resistant, harboring penicillin resistance (penicillin binding protein [*pbp1a*, *pbp2x*, and *pbp2b*]) and macrolide resistance (*mefA* and *ermB*) genes. Detailed drug susceptibility testing of the strain is shown in S1 Table. The minimum inhibitory concentration (MIC) of Penicillin G was 1, and therefore the strain was identified as clinically penicillin-intermediate *S*. *pneumoniae* (PISP) according to Clinical and Laboratory Standards Institute (CLSI) definition M100 S26:2016.

To establish the post-influenza secondary *S*. *pneumoniae* infection mouse model, mice were intranasally inoculated with five plaque-forming units (PFUs) of PR8. Seven days after PR8 infection, the mice were intranasally infected with 1 × 10^4^ colony-forming units (CFUs) of *S*. *pneumoniae* suspended in 50 μL of PBS. Mice were then intraperitoneally injected with CAM (100 mg/kg) diluted in PBS with 5% gum arabic or vehicle daily starting from 1 h after pneumococcal infection throughout the course of the experiment. Mice were monitored for survival for up to 5 days after pneumococcal infection. A 7-day interval between influenza infection and subsequent *S*. *pneumoniae* inoculation was used as a lethal model in the present study, based on previous studies [21, 57].

For IFN-γ administration, mice were intranasally inoculated with 16 μg/kg recombinant IFN-γ (PeproTech) or vehicle at 30 min and 24 h after pneumococcal infection. The dose of recombinant IFN-γ was determined according to a previous report [58] and survival was monitored for 7 days.

### Bronchoalveolar lavage

Bronchoalveolar lavage fluid (BALF) was collected with 0.7 mL of saline, and total cell counts were determined [59].

### Bacterial load

Whole left lungs were harvested and homogenized with 1 mL of sterile PBS with 0.1% Triton X and serially diluted with sterile PBS. Equal volumes of each dilution of the lung homogenates and blood were applied to trypticase soy agar (TSA) plates containing 5% sheep blood (BD Trypticase Soy Agar II with 5% Sheep Blood; BD Diagnostics) and incubated overnight at 37°C. After overnight incubation, bacterial colonies were counted and expressed as CFUs per mL of solution.

### Histological analysis

Lung specimens harvested 18 h after pneumococcal infection were fixed with 4% paraformaldehyde. After dehydration and embedding in paraffin, 3-μm sections were stained with hematoxylin and eosin (H&E).

### Protein extraction from whole lung tissues and immunoblotting

Whole lung tissues were homogenized using a Dounce tissue homogenizer on ice in RIPA Lysis and Extraction Buffer (Thermo Fisher Scientific). Homogenates were centrifuged at 12,000 × *g* at 4°C for 20 min, and the supernatants were collected and stored at −80°C until use for protein analysis. Bicinchoninic acid (BCA) protein assays (Thermo Fisher Scientific) were used to measure the total protein concentration in lung homogenates.

Equal amounts (15–30 μg) of cell lysates were fractionated by sodium dodecyl sulfate-polyacrylamide gel electrophoresis (Bio-Rad Laboratories), and the proteins were transferred to a polyvinylidene fluoride membrane (Bio-Rad Laboratories). After overnight incubation with antibodies against prok2 (Millipore, Billerica, MA, USA), STAT3, or p-STAT3 (both from Cell Signaling Technology, Danvers, MA, USA), the membrane was counterstained with horseradish peroxidase-conjugated rabbit IgG antibody and visualized with enhanced chemiluminescence detection reagents (GE Healthcare). Images were analyzed using ImageJ 1.45v (National Institutes of Health, Bethesda, MD, USA).

### Analysis of anti-Bv8/Prok2 antibody treatment

The anti-Bv8 antibody was kindly provided by Genentech. Mice were intraperitoneally injected with the anti-Bv8 antibody (5 mg/kg) or control IgG at 4 days and 1 day before CAM treatment and once daily for three consecutive days during CAM treatment.

### Quantitative real-time polymerase chain reaction

Total RNA was isolated from the lung or spleen using the RNeasy Mini Kit according to the manufacturer’s instructions (QIAGEN). Total RNA was reverse-transcribed using the High-Capacity RNA-to-cDNA cDNA Kit (Life Technologies). Real-time quantitative polymerase chain reaction (PCR) was performed using SYBR Green chemistry on a 7500 Fast Real-Time PCR system (Applied Biosystems). The primer sequences for each target gene are provided in S2 Table.

### Purification of human peripheral blood cells

Peripheral blood cells were isolated from healthy subjects. Red blood cells (RBCs) were removed from 40 mL of heparinized peripheral blood using Dextran T-500 (Pfizer). After removal any remaining RBC using ammonium-chloride-potassium (ACK) lysis buffer (Lonza, Basel, Switzerland), peripheral cells were stained with antibodies described above.

### Statistical analysis

Data are expressed as the mean ± SEM and analyzed using the Mann–Whitney-U-test, Wilcoxon matched-pairs signed rank test, or ANOVA, followed by Tukey’s tests for multiple comparisons. Log-rank tests were performed for survival studies. *p* < 0.05 was considered statistically significant.

## Supporting information

S1 Table Drug susceptibility of the *S*. *pneumoniae* strain used in the present study. Minimum inhibitory concentrations of indicated antimicrobial agents are shown.
(DOCX)

S2 Table Primer sequences used in quantitative real-time PCR. Primer sequences of *Arg1*, *Il10*, *Prok2*, *and ARG1* are shown.
(DOCX)

S1 Fig Clarithromycin expands CD11b^+^Gr-1^+^ cells in a dose- and time-dependent manner. (A and B) Mice were intraperitoneally injected with CAM once a day with doses of 0 (vehicle control), 20 mg/kg, 50 mg/kg, 100 mg/kg, and 200 mg/kg for three consecutive days. On the day after the last injection, CD11b^+^Gr-1^+^ cells in the spleen (A) and lungs (B) were analyzed by flow cytometry (n = 4 in each condition). (C and D) Mice were intraperitoneally injected with either CAM (100 mg/kg) or vehicle daily, starting from day 0 through the day before the indicated days. CD11b^+^Gr-1^+^ cells in the spleen (C) and lungs (D) were then analyzed by flow cytometry (n = 4 in each condition). Data are presented as the mean ± SEM. *p < 0.05; **p < 0.01; ***p < 0.001 by the Mann–Whitney U-tests.
(TIF)

S2 Fig Immunofluorescence staining and FACS analysis of Gr-1^+^ cells in lungs. (A and B) Gr-1 immunofluorescence staining in the lungs of mice treated with (A) vehicle or (B) CAM daily for three consecutive days (n = 4 per group). Scale bar, 100 μm. (C) Two-parameter dot plots of CD11b^+^Gr-1^+^ cells in lungs sorted from mice intraperitoneally treated with vehicle or CAM daily for three consecutive days. The mice were intravenously injected with an APC-Cy7-CD45 antibody conjugate for 5 min, sacrificed, and intratracheally injected with a PerCP-Cy5.5-CD45 antibody conjugate for 5 min. Next, a lung single cell suspension was prepared and stained with a PE-Cy7-CD45 antibody conjugate.
(TIF)

S3 Fig Arginase-1 mRNA expression after intraperitoneal and oral CAM administration. (A) Mice were intraperitoneally administered CAM daily for three consecutive days. On the day after the last administration, splenic CD11b^+^Gr-1^+^ cells were sorted and arginase-1 mRNA (*Arg1*) expression was measured by quantitative real-time PCR. (B) Mice were orally administered CAM daily for seven consecutive days. The day after the last administration, splenic CD11b^+^Gr-1^+^ cells were sorted and *Arg1* expression was measured by quantitative real-time PCR (n = 3 in each group). Data are presented as the mean ± SEM.
(TIF)

S4 Fig Elastase activity, MPO activity, and phagocytic activity in CAM-treated CD11b^+^Gr-1^+^ cells. (A) Elastase activity in vehicle-treated CD11b^+^Gr-1^+^ cells (a), CAM-treated CD11b^+^Gr-1^+^ cells (b), LPS-treated CD11b^+^Gr-1^+^ cells (c), thioglycolate-induced neutrophils (d), and isolated peripheral neutrophils (e) was measured using the commercially available Neutrophil Elastase Activity Assay Kit (n = 3). (B) MPO activity in indicated cells was measured using the commercially available MPO Activity Assay Kit (n = 3). (C) Phagocytic activity in indicated cells was measured using the commercially available Phagocytosis Activity Assay Kit (n = 3). f: Isolated monocytes.
(TIF)

S5 Fig CD3^+^ T cell proliferation assay after co-culture with vehicle-treated or CAM-treated CD11b^+^Gr-1^+^ cells. CD3^+^ T cell proliferation was measured by the carboxyfluorescein succinimidyl ester (CFSE) method when co-cultured with equal numbers of vehicle-treated or CAM-treated CD11b^+^Gr-1^+^ cells (1 × 10^5^ cells) from the spleen. (n = 4 per group). A representative histogram is shown.
(TIF)

S6 Fig Surface expression of various immune markers in CAM-treated CD11b^+^Gr-1^+^ cells. Various surface markers, including CD244, CTLA-4, PD-1, PD-L1, CXCR2, CXCR4, CD80, CD115, and CX3CR1, on splenic CD11b^+^Gr-1^+^ cells were measured by flow cytometry (n = 4 per group).
(TIF)

S7 Fig Potency of CAM and other macrolides in the expansion of CD11b^+^Gr-1^+^ cells. (A) Mice were intraperitoneally injected with vehicle, clarithromycin (CAM) (100 mg/kg), azithromycin (AZM) (100 mg/kg), or josamycin (JOS) (200 mg/kg) daily for three consecutive days. Representative two-parameter dot plots of CD11b^+^Gr-1^+^ cells in the spleen (upper panel) and lungs (lower panel) are shown. (B and C) Quantification of splenic (B) and lung (C) CD11b^+^Gr-1^+^ cells obtained from vehicle-, CAM-, AZM-, and JOS-treated mice are shown (n = 8–9 in each group). N.S., not significant. **p < 0.01; ***p < 0.001; #*p* < 0.05; ###*p* < 0.001 by a one-way ANOVA with Tukey’s multiple comparison tests.
(TIF)

S8 Fig Experimental schema for depletion of the Gr-1^+^ cell population. (A) Pharmacological depletion of the Gr-1^+^ cell population using an anti-Gr-1 antibody was performed 24 h before LPS challenge (results summarized in Fig 3H). (B) Pharmacological depletion of the Gr-1^+^ cell population using an anti-Gr-1 antibody was performed 1 h before initiation of CAM treatment (i.e., 73 h before LPS challenge) (results summarized in Fig 3I).
(TIF)

S9 Fig Adoptive transfer of CAM- and vehicle-treated CD11b^+^Gr-1^+^ cells, and PBS control injection in LPS endotoxin shock. CAM- and vehicle-treated CD11b^+^Gr-1^+^ cells (1 × 10^6^ cells) from the spleen and PBS control were intravenously injected via tail vein in mice subjected to LPS endotoxin shock. (n = 15–16 per group). N.S.; not significant by the log-rank test.
(TIF)

S10 Fig Expansion of CD11^+^Gr-1^+^ cells is independent of IL-10. WT and *Il10*^*-/-*^ mice were intraperitoneally injected with vehicle or clarithromycin (CAM) (100 mg/kg) daily for three consecutive days. On the day after the last injection, single splenic and lung cell suspensions were subjected to flow cytometry. Representative two-parameter dot plots of CD11b^+^Gr-1^+^ cells from the spleen are shown (n = 3 in each condition).
(TIF)

S11 Fig Josamycin does not improve survival in post-influenza pneumococcal pneumonia. Survival rate of mice with post-influenza pneumococcal pneumonia treated with vehicle, clarithromycin (CAM) (100 mg/kg), or josamycin (JOS) (200 mg/kg) (n = 15–16 per group). N.S.; not significant. *p < 0.05 by the log-rank test.
(TIF)

S12 Fig Role of type I IFN in post-influenza pneumococcal pneumonia. Survival rate of vehicle- or CAM-treated (100 mg/kg daily for consecutive days) WT and *Ifnar1*^*-/-*^ mice in post-influenza pneumococcal pneumonia mice (n = 4–16 per group).
(TIF)

S13 Fig CD11^+^Gr-1^+^ cell expansion is independent of IL-17, CCL2, MyD88, and CXCR4. (A–C) WT, *Il17a*^*-/-*^ mice (A), *Ccl2*^*-/-*^ mice (B), or *Myd88*^*-/-*^ mice (C) were intraperitoneally injected with vehicle or clarithromycin (CAM) (100 mg/kg) daily for three consecutive days. On the day after the last injection, single splenic cell suspensions were subjected to flow cytometry. Representative two-parameter dot plots of CD11b^+^Gr-1^+^ cells from the spleen are shown (n = 4 in each condition). (D) Mice were intraperitoneally treated with a CXCR4 antagonist (AMD3100, Millipore) (5 mg/kg) or vehicle. One hour after injection, mice were intraperitoneally injected with vehicle or CAM (100 mg/kg) daily for three consecutive days, as described in (A–C). Representative two-parameter dot plots of splenic CD11b^+^Gr-1^+^ cells are shown (n = 4 in each condition).
(TIF)

S14 Fig Gut commensal microbiota may be involved CD11b^+^Gr-1^+^ cell expansion. (A and B) Representative two-parameter dot plots of CD11b^+^Gr-1^+^ cells in the spleen (A) and lungs (B) of mice treated with vehicle or CAM (100 mg/day) for three consecutive days in specific pathogen-free (SPF) mice and germ-free mice (n = 3 per group). (C) Percentage of CD11b^+^Gr-1^+^ cells in the spleen and lungs of mice treated with vehicle or CAM (100 mg/day) for three consecutive days in SPF mice and germ-free mice. *p < 0.05 (n = 3 per group). (D and E) Quantification of CD11b^+^Gr-1^+^ cells in the spleen (D) and lungs (E) of SPF and germ-free mice treated with vehicle or CAM (100 mg/day) treatment for three consecutive days. *p < 0.05 (n = 3 per group).
(TIF)

S15 Fig Schematic illustration of the immunosuppressive effects of CAM-treated CD11b^+^Gr-1^+^ cells in the present study. CAM expands the CD11b^+^Gr-1^+^ cell population depending on the STAT3/Bv8 signaling pathway. CAM-treated CD11b^+^Gr-1^+^ cells subsequently protect mice against LPS-induced shock mostly via increased IL-10, and protect mice from post-influenza pneumococcal pneumonia, mainly via decreased IFN-γ.
(TIF)
