# Supplementary material for: Clarithromycin expands CD11b+Gr-1+ cells via the STAT3/Bv8 axis to ameliorate lethal endotoxic shock and post-influenza bacterial pneumonia
Source: PLoS Pathog. 2018 Apr 5;14(4):e1006955. doi: 10.1371/journal.ppat.1006955 (PMC5886688; doi:10.1371/journal.ppat.1006955)
Supplement: S1 Table — Minimum inhibitory concentrations of indicated antimicrobial agents are shown. (DOCX) [file ppat.1006955.s001.docx]

**S1 Table. Drug susceptibility of the *S. pneumoniae* strain used in the present study**

| **Antimicrobial agent** | **Minimum inhibitory concentration (MIC, μg/mL)** |
| --- | --- |
| Tebipenem | 0.125 |
| Penicillin G | 1 |
| Ampicillin | 2 |
| Amoxicillin | 1 |
| Ceftriaxone | 1 |
| Panipenem | 0.125 |
| Meropenem | 1 |
| Levofloxacin | 1 |
| Vancomycin | 0.5 |
| Clarithromycin | 64 |
