# Supplementary material for: Clarithromycin expands CD11b+Gr-1+ cells via the STAT3/Bv8 axis to ameliorate lethal endotoxic shock and post-influenza bacterial pneumonia
Source: PLoS Pathog. 2018 Apr 5;14(4):e1006955. doi: 10.1371/journal.ppat.1006955 (PMC5886688; doi:10.1371/journal.ppat.1006955)
Supplement: S2 Table — Primer sequences of Arg1, Il10, Prok2, and ARG1 are shown. (DOCX) [file ppat.1006955.s002.docx]

**S2 Table. Primer sequences used in quantitative real-time PCR**

| **Gene** | **Forward (5'-3')** | **Reverse (5'-3')** |
| --- | --- | --- |
| *Arg1* | ATGGAAGAGACCTTCAGCTAC | GCTGTCTTCCCAAGAGTTGGG |
| *Il10* | CTTCGAGATCTCCGAGATGCCTTC | ATTCTTCACCTGCTCCACGGCCTT |
| *Prok2* | CTTCGCCCTTCTTCTTTCCT | GCATGTGCTGTGCTGTCAGT |
| *ARG1* | acagtttggcaattggaagca | cacccagatgactccaagatcag |
