# Supplementary figures and images for: Clarithromycin expands CD11b+Gr-1+ cells via the STAT3/Bv8 axis to ameliorate lethal endotoxic shock and post-influenza bacterial pneumonia
Source: PLoS Pathog. 2018 Apr 5;14(4):e1006955. doi: 10.1371/journal.ppat.1006955 (PMC5886688; doi:10.1371/journal.ppat.1006955)

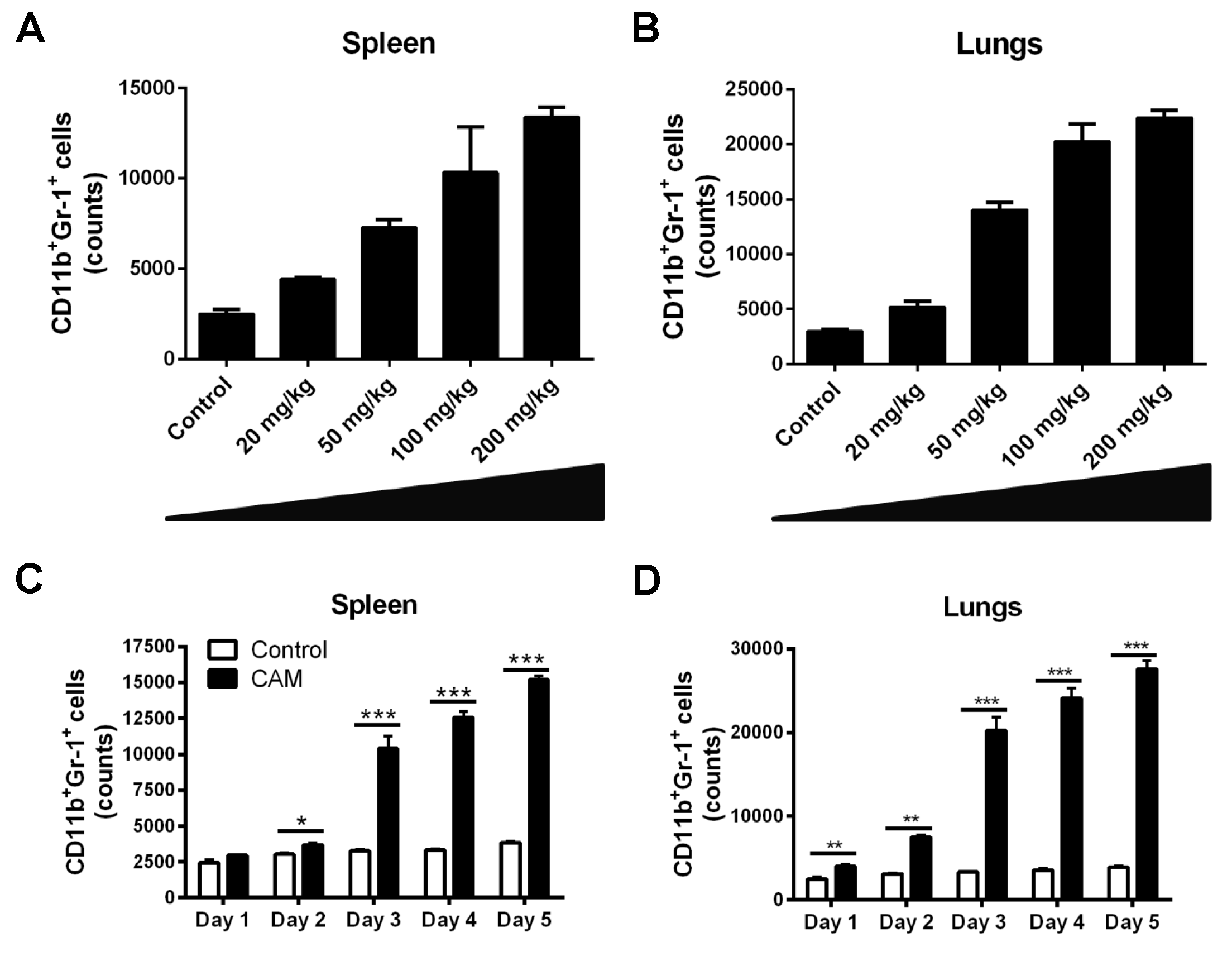

Supplement: S1 Fig — (A and B) Mice were intraperitoneally injected with CAM once a day with doses of 0 (vehicle control), 20 mg/kg, 50 mg/kg, 100 mg/kg, and 200 mg/kg for three consecutive days. On the day after the last injection, CD11b+Gr-1+ cells in the spleen (A) and lungs (B) were analyzed by flow cytometry (n = 4 in each condition). (C and D) Mice were intraperitoneally injected with either CAM (100 mg/kg) or vehicle daily, starting from day 0 through the day before the indicated days. CD11b+Gr-1+ cells in the spleen (C) and lungs (D) were then analyzed by flow cytometry (n = 4 in each condition). Data are presented as the mean ± SEM. *p < 0.05; **p < 0.01; ***p < 0.001 by the Mann–Whitney U-tests. (TIF) [file ppat.1006955.s003.tif]

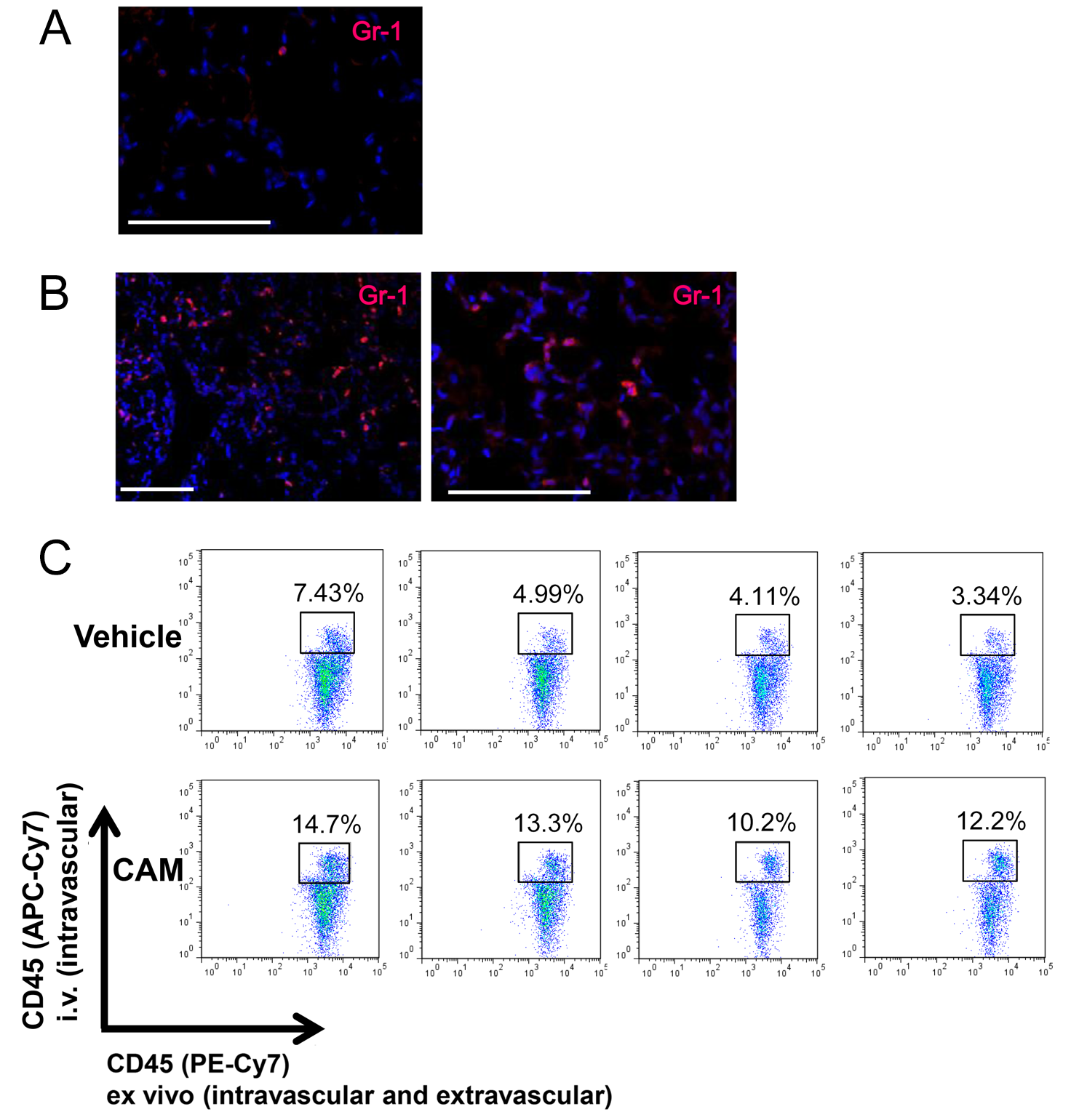

Supplement: S2 Fig — (A and B) Gr-1 immunofluorescence staining in the lungs of mice treated with (A) vehicle or (B) CAM daily for three consecutive days (n = 4 per group). Scale bar, 100 μm. (C) Two-parameter dot plots of CD11b+Gr-1+ cells in lungs sorted from mice intraperitoneally treated with vehicle or CAM daily for three consecutive days. The mice were intravenously injected with an APC-Cy7-CD45 antibody conjugate for 5 min, sacrificed, and intratracheally injected with a PerCP-Cy5.5-CD45 antibody conjugate for 5 min. Next, a lung single cell suspension was prepared and stained with a PE-Cy7-CD45 antibody conjugate. (TIF) [file ppat.1006955.s004.tif]

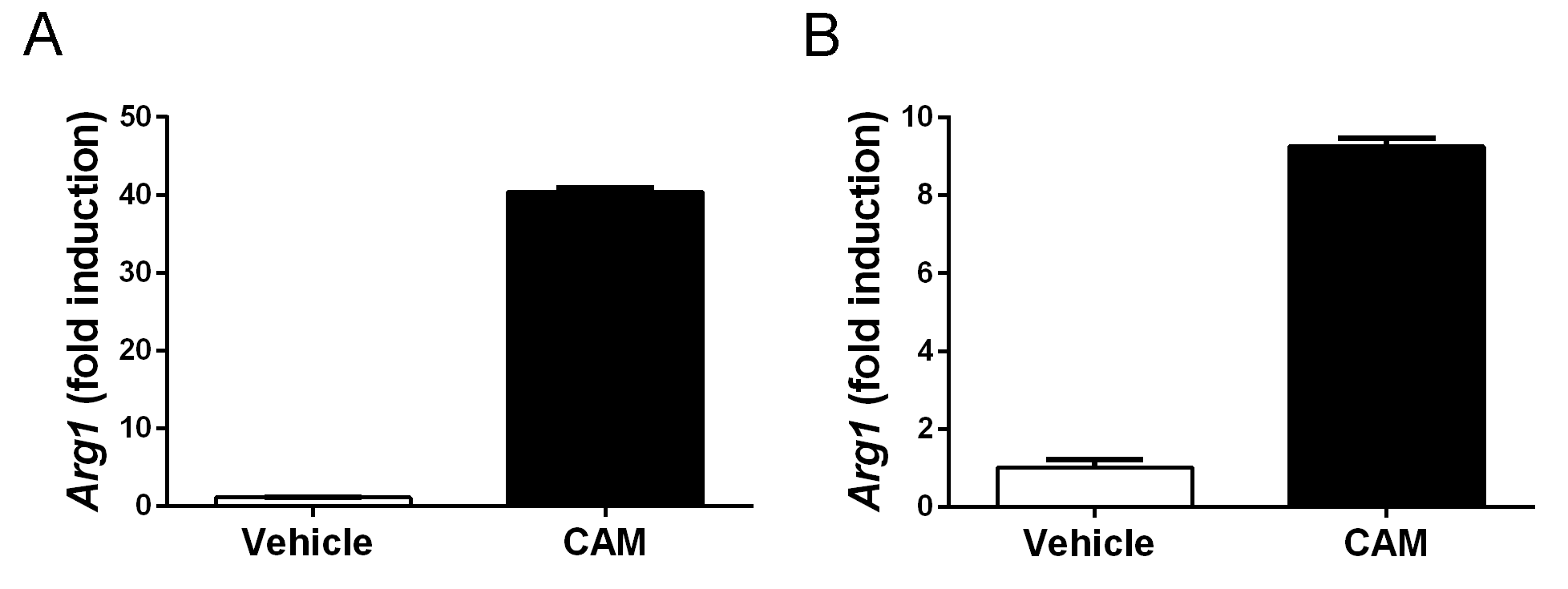

Supplement: S3 Fig — (A) Mice were intraperitoneally administered CAM daily for three consecutive days. On the day after the last administration, splenic CD11b+Gr-1+ cells were sorted and arginase-1 mRNA (Arg1) expression was measured by quantitative real-time PCR. (B) Mice were orally administered CAM daily for seven consecutive days. The day after the last administration, splenic CD11b+Gr-1+ cells were sorted and Arg1 expression was measured by quantitative real-time PCR (n = 3 in each group). Data are presented as the mean ± SEM. (TIF) [file ppat.1006955.s005.tif]

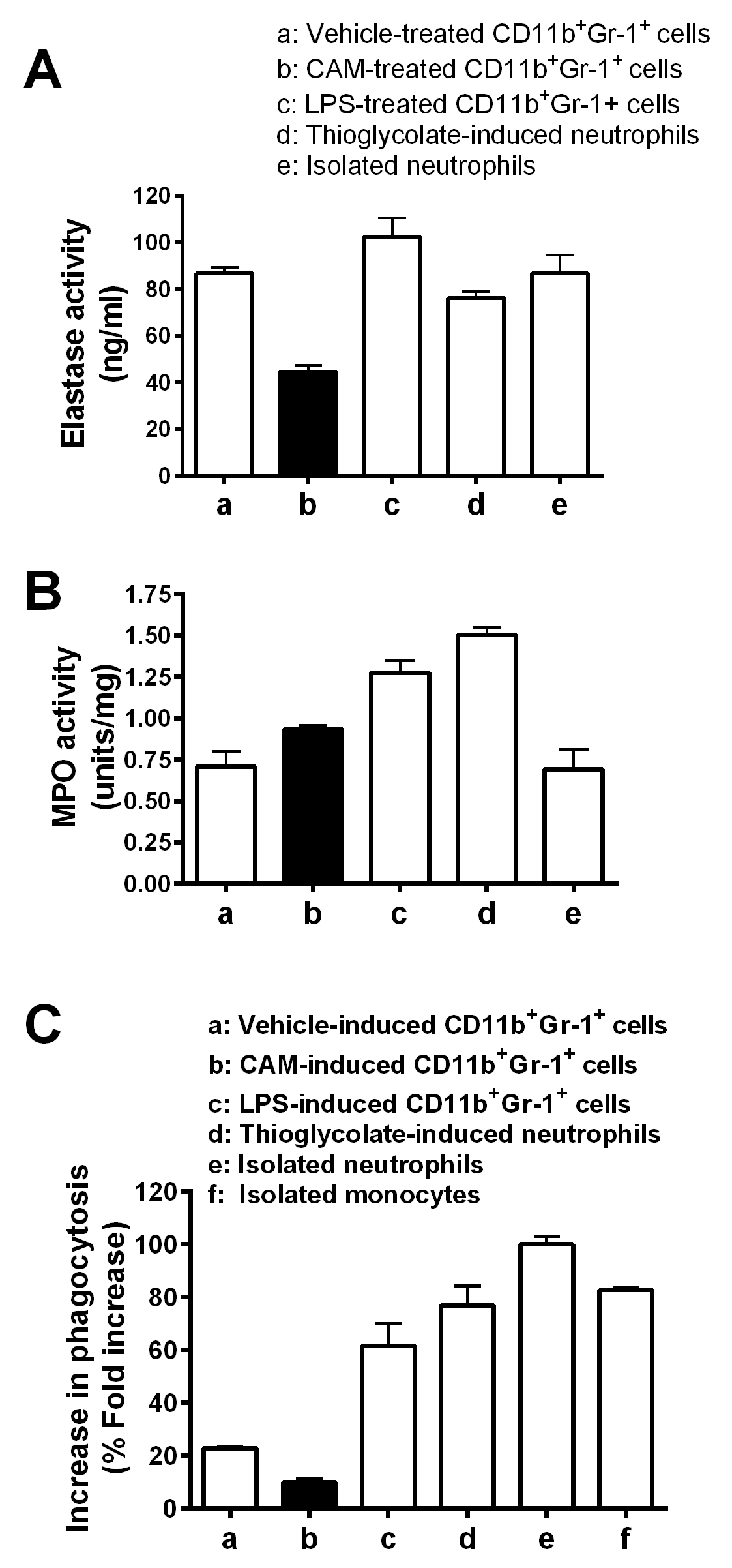

Supplement: S4 Fig — (A) Elastase activity in vehicle-treated CD11b+Gr-1+ cells (a), CAM-treated CD11b+Gr-1+ cells (b), LPS-treated CD11b+Gr-1+ cells (c), thioglycolate-induced neutrophils (d), and isolated peripheral neutrophils (e) was measured using the commercially available Neutrophil Elastase Activity Assay Kit (n = 3). (B) MPO activity in indicated cells was measured using the commercially available MPO Activity Assay Kit (n = 3). (C) Phagocytic activity in indicated cells was measured using the commercially available Phagocytosis Activity Assay Kit (n = 3). f: Isolated monocytes. (TIF) [file ppat.1006955.s006.tif]

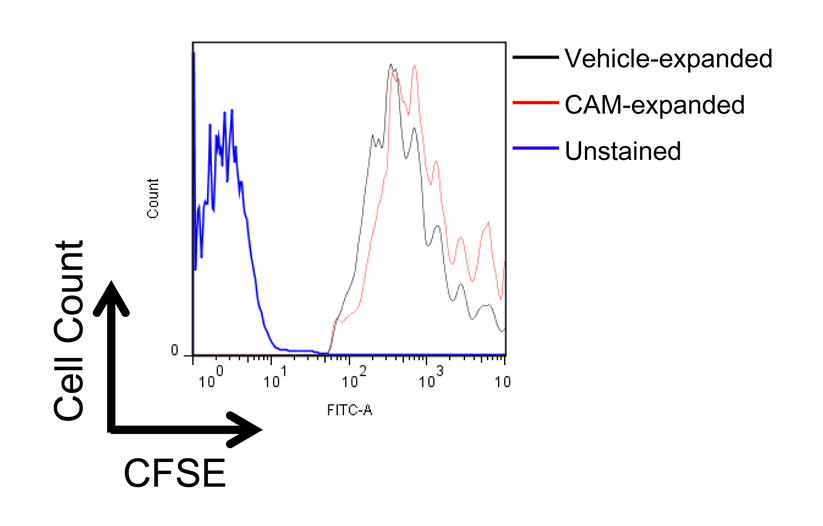

Supplement: S5 Fig — CD3+ T cell proliferation was measured by the carboxyfluorescein succinimidyl ester (CFSE) method when co-cultured with equal numbers of vehicle-treated or CAM-treated CD11b+Gr-1+ cells (1 × 10^5^ cells) from the spleen. (n = 4 per group). A representative histogram is shown. (TIF) [file ppat.1006955.s007.tif]

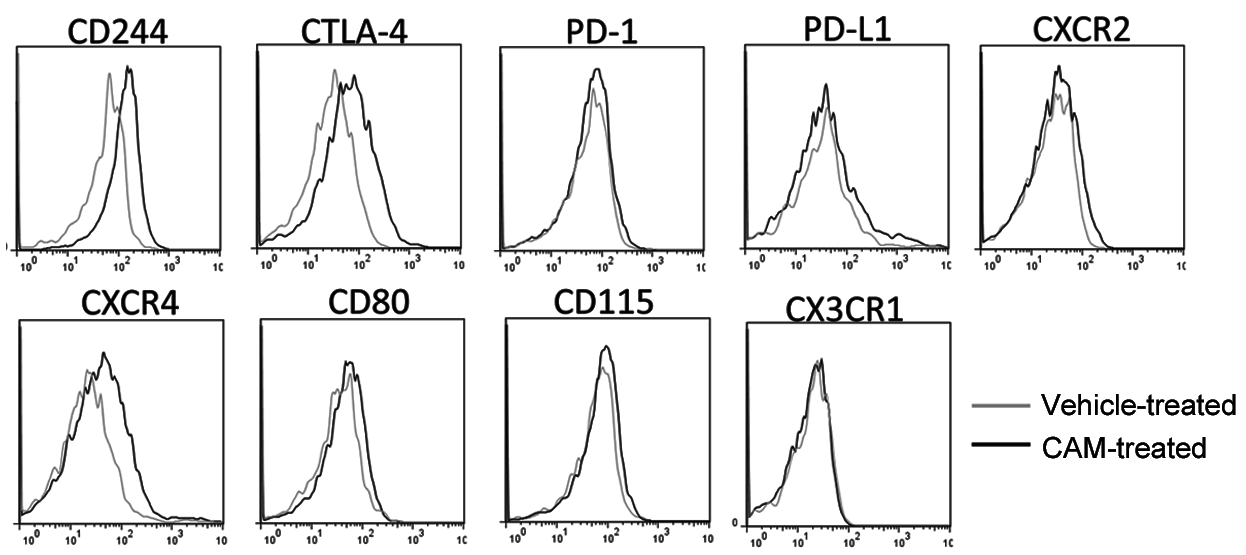

Supplement: S6 Fig — Various surface markers, including CD244, CTLA-4, PD-1, PD-L1, CXCR2, CXCR4, CD80, CD115, and CX3CR1, on splenic CD11b+Gr-1+ cells were measured by flow cytometry (n = 4 per group). (TIF) [file ppat.1006955.s008.tif]

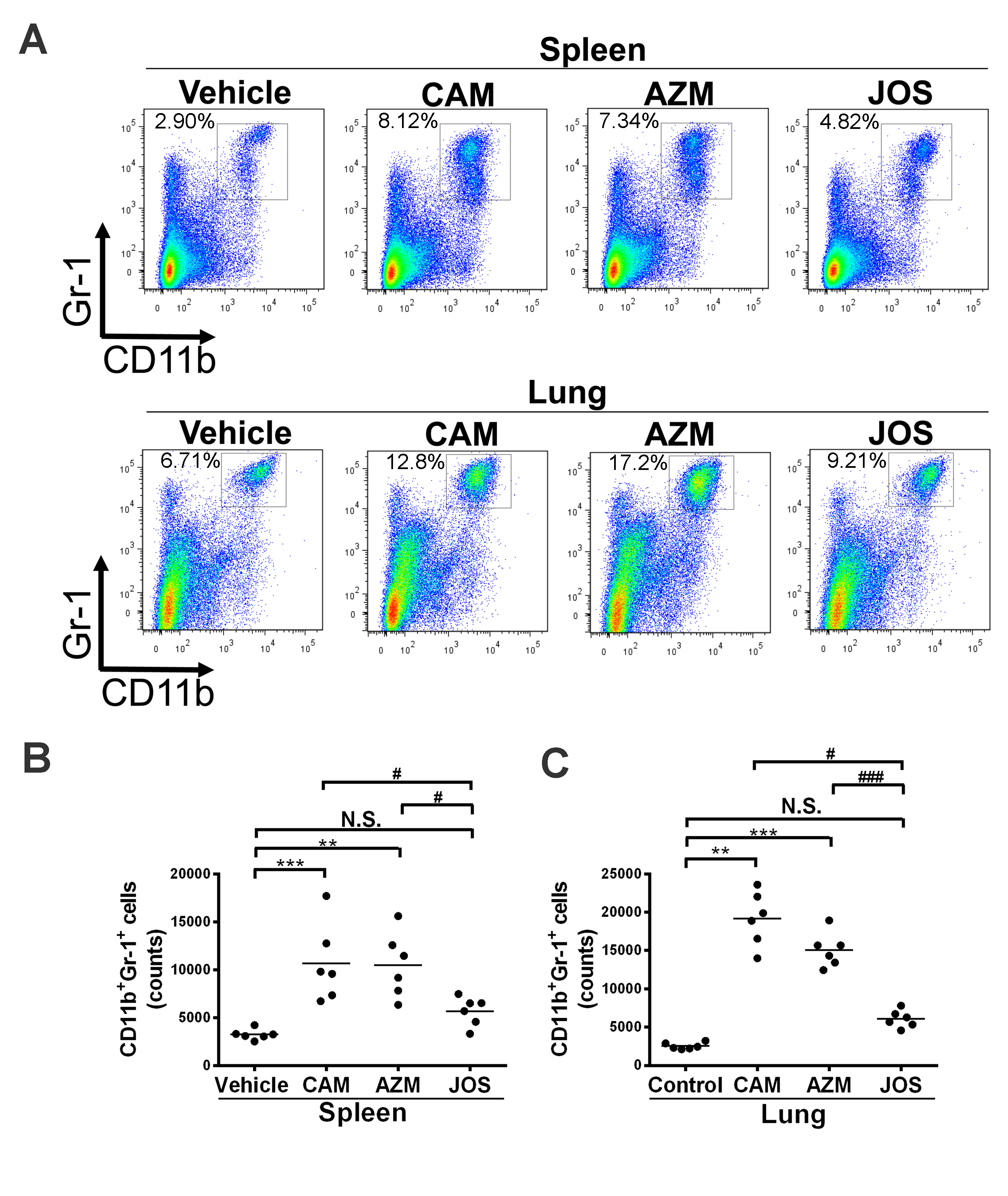

Supplement: S7 Fig — (A) Mice were intraperitoneally injected with vehicle, clarithromycin (CAM) (100 mg/kg), azithromycin (AZM) (100 mg/kg), or josamycin (JOS) (200 mg/kg) daily for three consecutive days. Representative two-parameter dot plots of CD11b+Gr-1+ cells in the spleen (upper panel) and lungs (lower panel) are shown. (B and C) Quantification of splenic (B) and lung (C) CD11b+Gr-1+ cells obtained from vehicle-, CAM-, AZM-, and JOS-treated mice are shown (n = 8–9 in each group). N.S., not significant. **p < 0.01; ***p < 0.001; #p < 0.05; ###p < 0.001 by a one-way ANOVA with Tukey’s multiple comparison tests. (TIF) [file ppat.1006955.s009.tif]

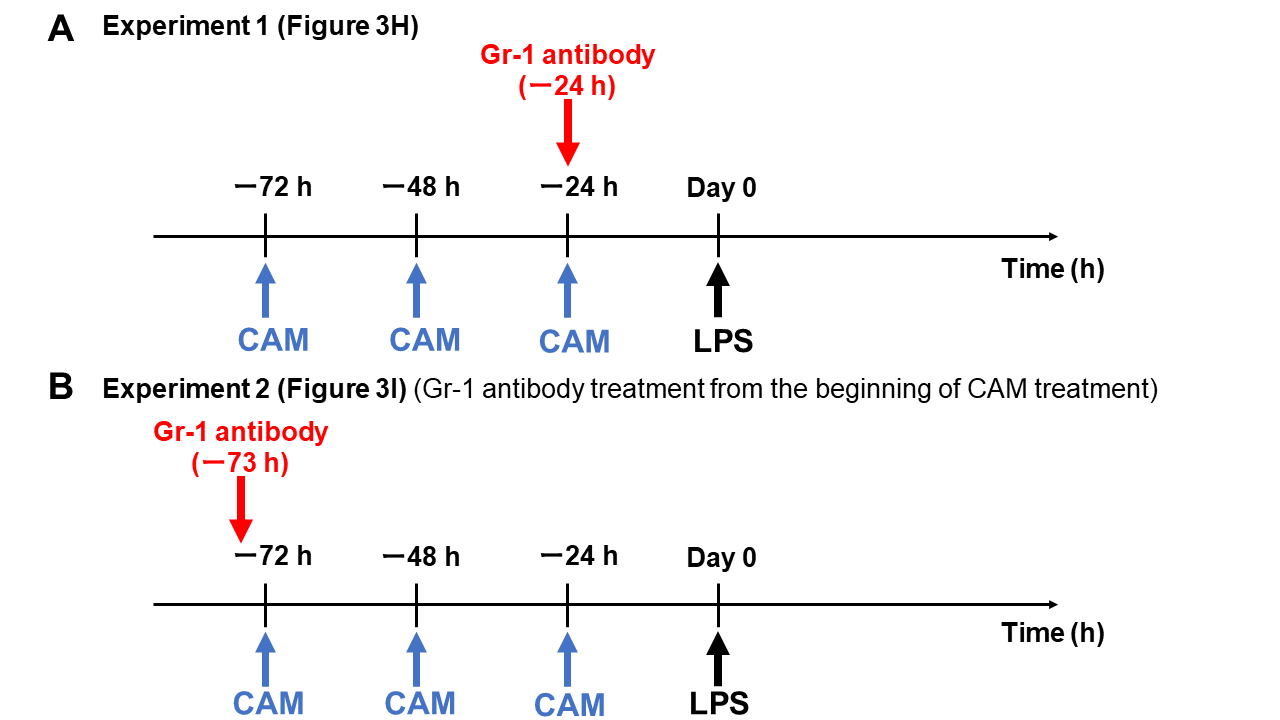

Supplement: S8 Fig — (A) Pharmacological depletion of the Gr-1+ cell population using an anti-Gr-1 antibody was performed 24 h before LPS challenge (results summarized in Fig 3H). (B) Pharmacological depletion of the Gr-1+ cell population using an anti-Gr-1 antibody was performed 1 h before initiation of CAM treatment (i.e., 73 h before LPS challenge) (results summarized in Fig 3I). (TIF) [file ppat.1006955.s010.tif]

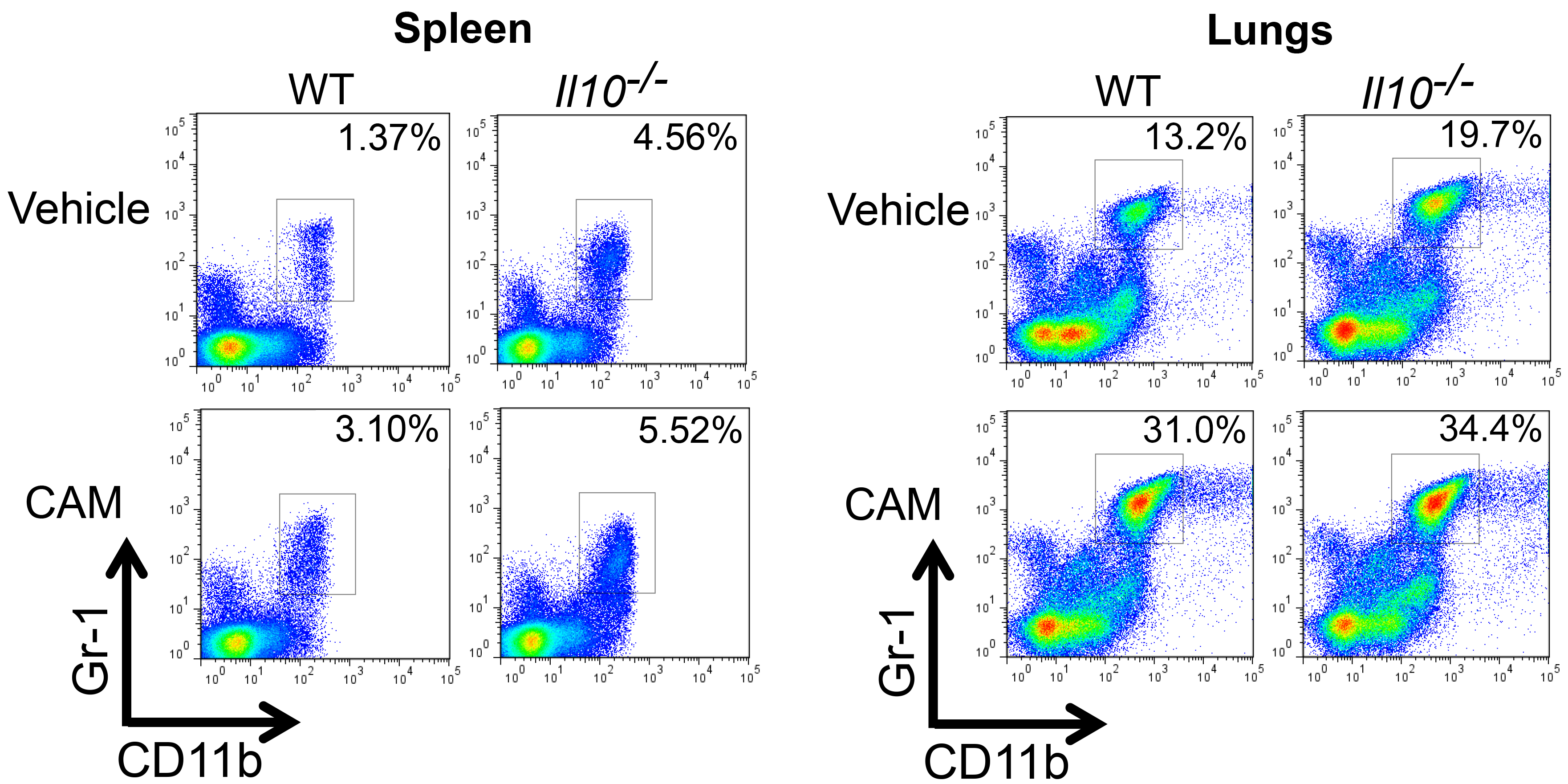

Supplement: S10 Fig — WT and Il10-/- mice were intraperitoneally injected with vehicle or clarithromycin (CAM) (100 mg/kg) daily for three consecutive days. On the day after the last injection, single splenic and lung cell suspensions were subjected to flow cytometry. Representative two-parameter dot plots of CD11b+Gr-1+ cells from the spleen are shown (n = 3 in each condition). (TIF) [file ppat.1006955.s012.tif]

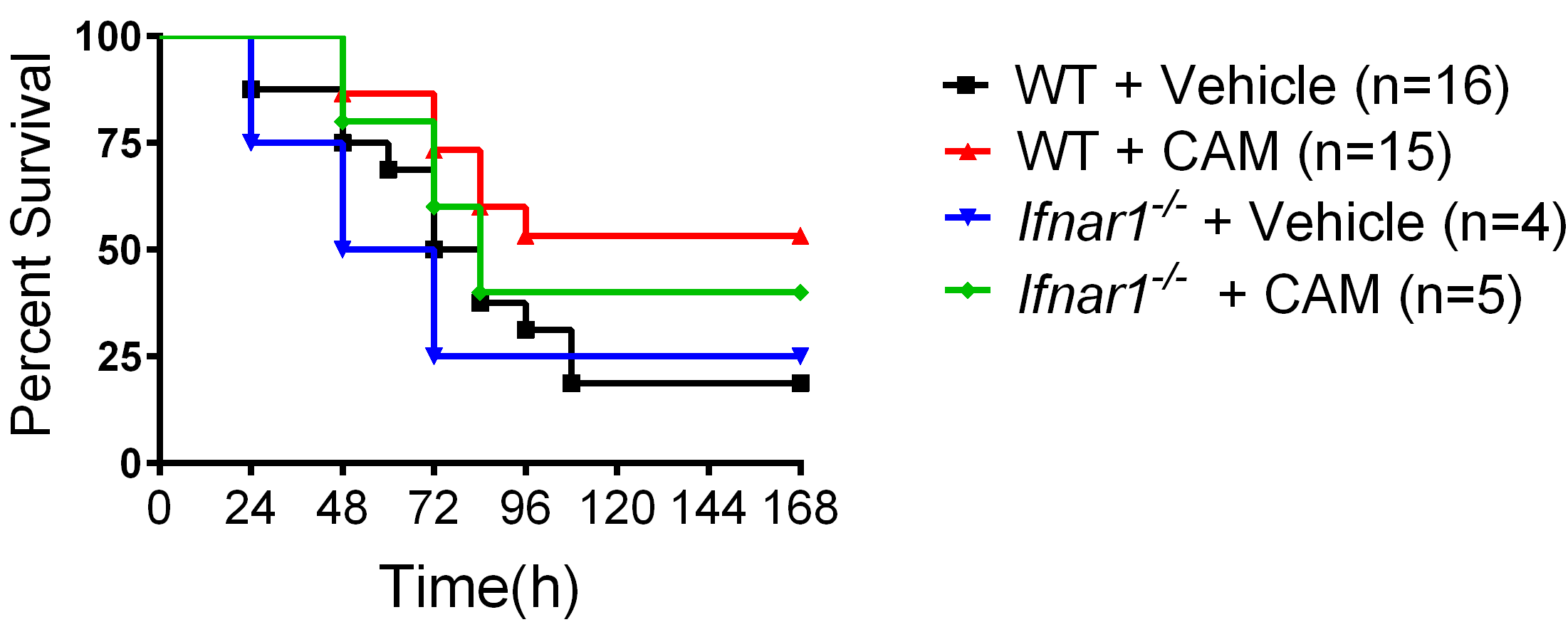

Supplement: S12 Fig — Survival rate of vehicle- or CAM-treated (100 mg/kg daily for consecutive days) WT and Ifnar1-/- mice in post-influenza pneumococcal pneumonia mice (n = 4–16 per group). (TIF) [file ppat.1006955.s014.tif]

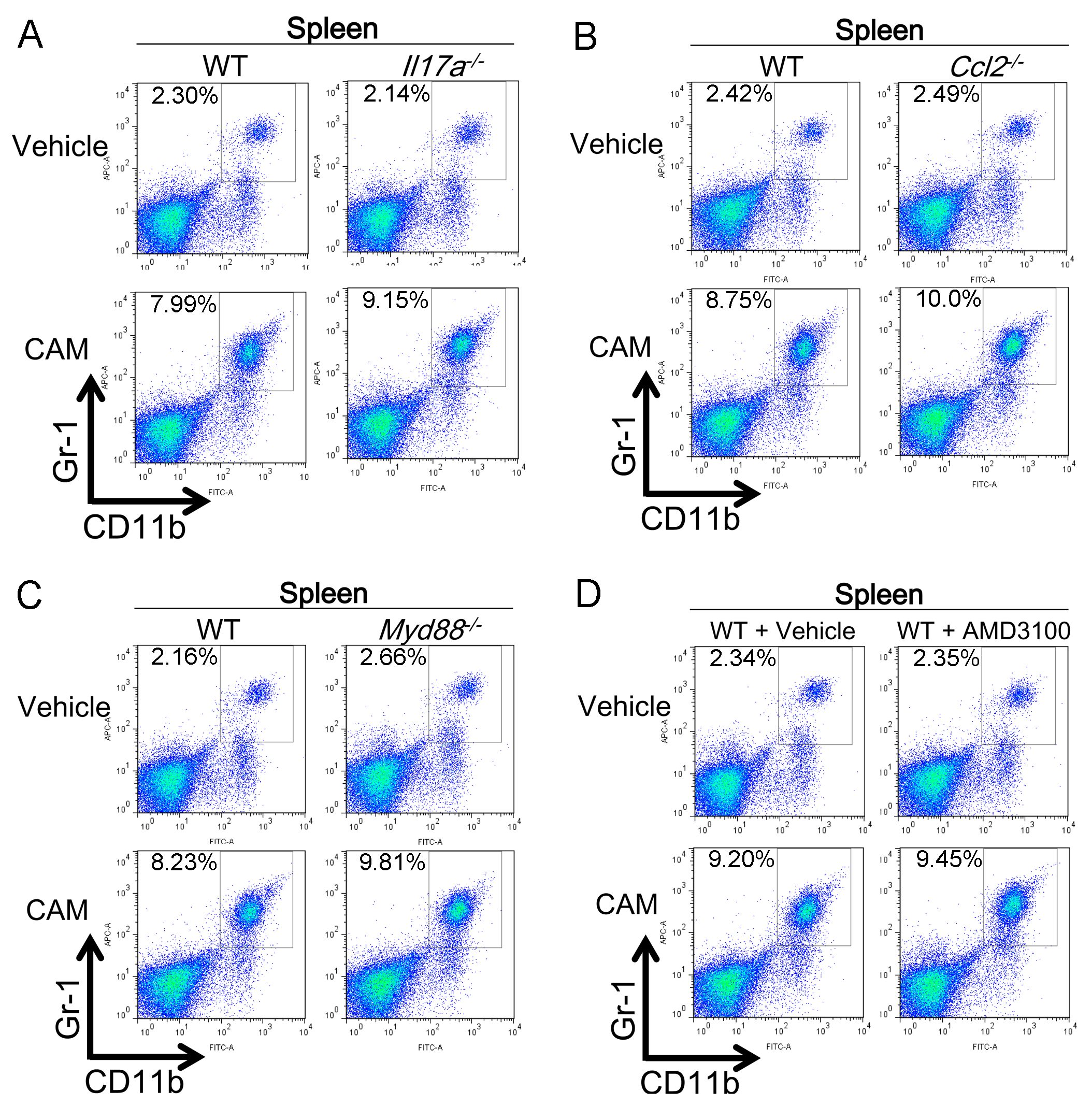

Supplement: S13 Fig — (A–C) WT, Il17a-/- mice (A), Ccl2-/- mice (B), or Myd88-/- mice (C) were intraperitoneally injected with vehicle or clarithromycin (CAM) (100 mg/kg) daily for three consecutive days. On the day after the last injection, single splenic cell suspensions were subjected to flow cytometry. Representative two-parameter dot plots of CD11b+Gr-1+ cells from the spleen are shown (n = 4 in each condition). (D) Mice were intraperitoneally treated with a CXCR4 antagonist (AMD3100, Millipore) (5 mg/kg) or vehicle. One hour after injection, mice were intraperitoneally injected with vehicle or CAM (100 mg/kg) daily for three consecutive days, as described in (A–C). Representative two-parameter dot plots of splenic CD11b+Gr-1+ cells are shown (n = 4 in each condition). (TIF) [file ppat.1006955.s015.tif]

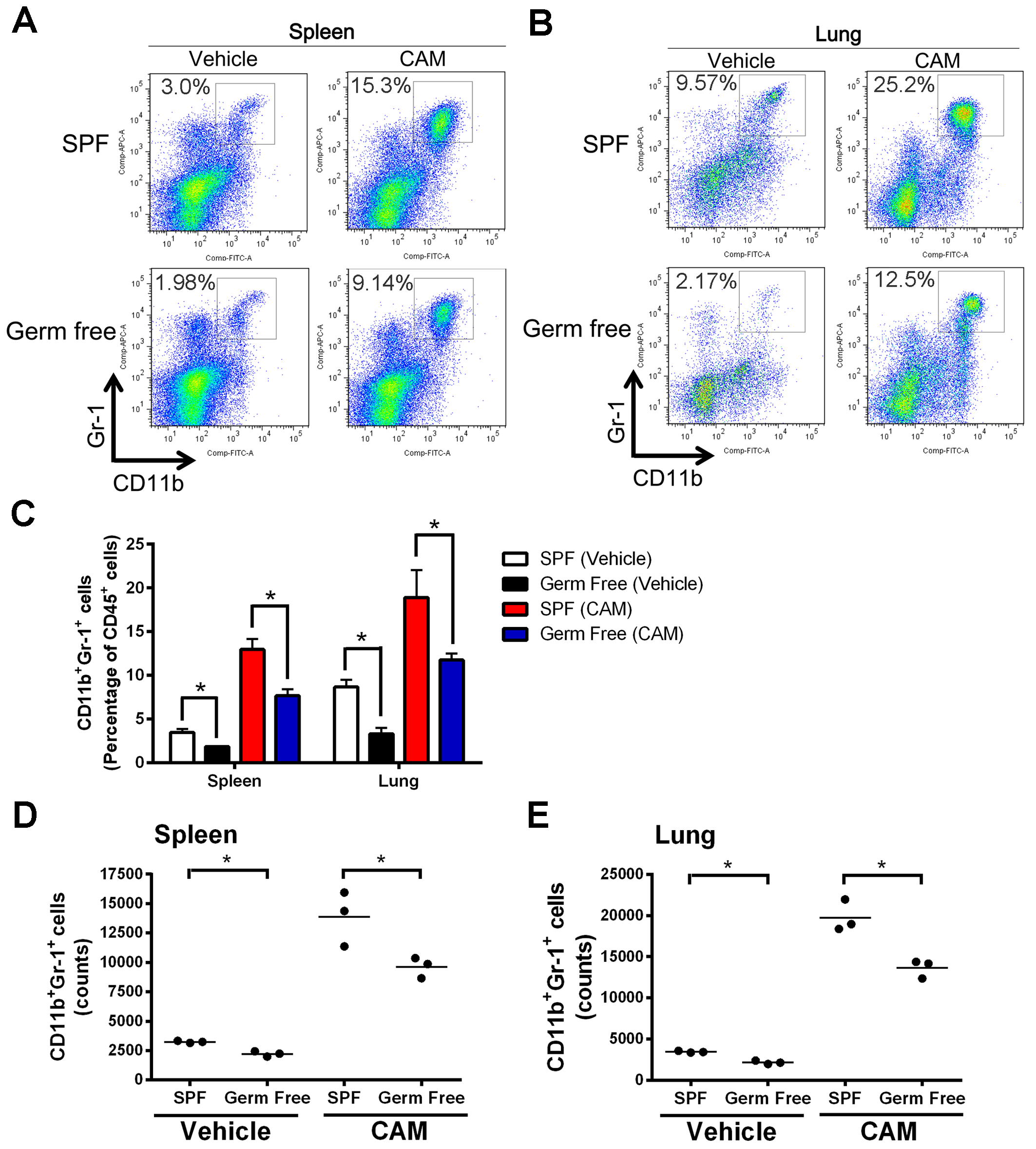

Supplement: S14 Fig — (A and B) Representative two-parameter dot plots of CD11b+Gr-1+ cells in the spleen (A) and lungs (B) of mice treated with vehicle or CAM (100 mg/day) for three consecutive days in specific pathogen-free (SPF) mice and germ-free mice (n = 3 per group). (C) Percentage of CD11b+Gr-1+ cells in the spleen and lungs of mice treated with vehicle or CAM (100 mg/day) for three consecutive days in SPF mice and germ-free mice. *p < 0.05 (n = 3 per group). (D and E) Quantification of CD11b+Gr-1+ cells in the spleen (D) and lungs (E) of SPF and germ-free mice treated with vehicle or CAM (100 mg/day) treatment for three consecutive days. *p < 0.05 (n = 3 per group). (TIF) [file ppat.1006955.s016.tif]

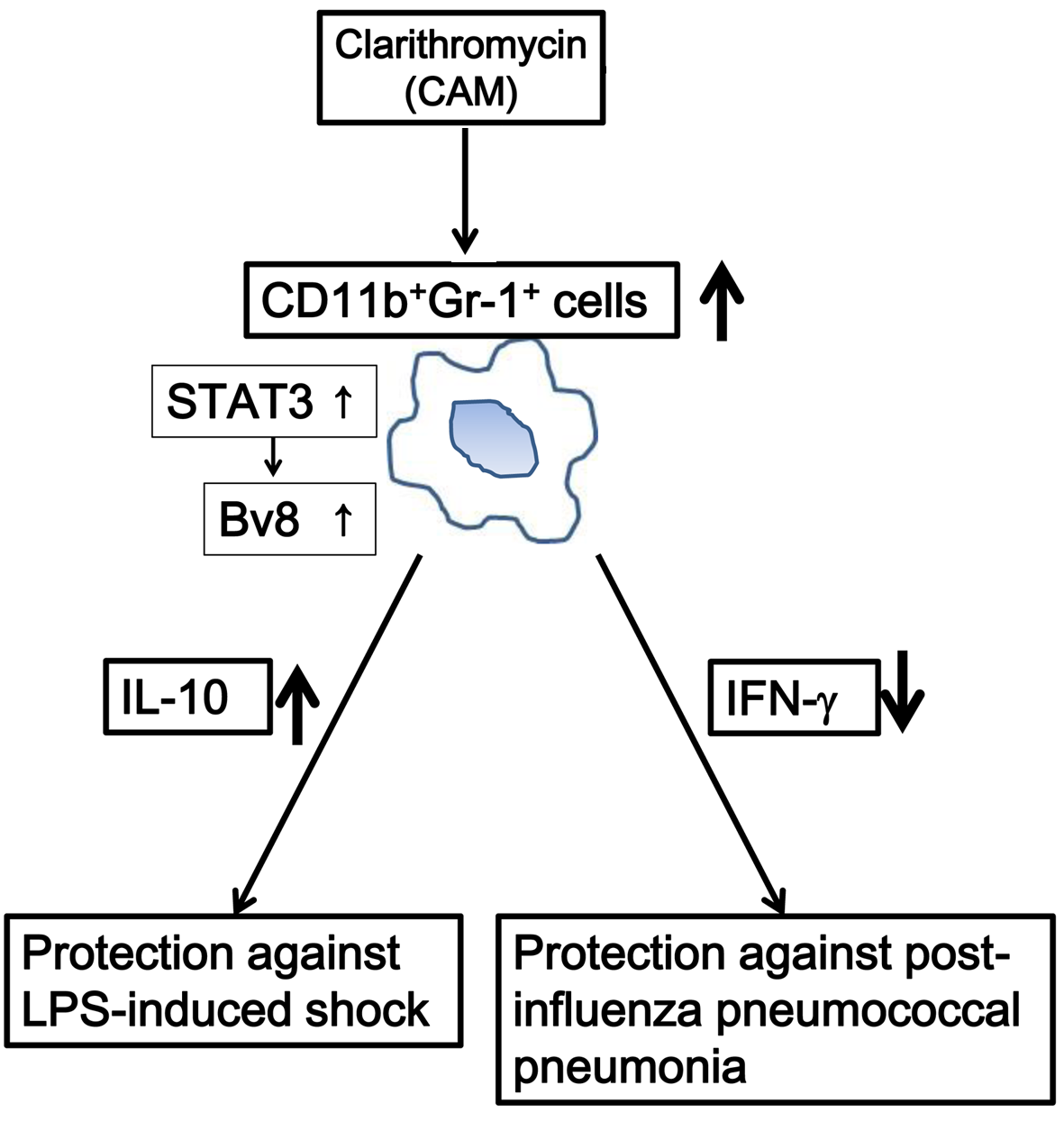

Supplement: S15 Fig — CAM expands the CD11b+Gr-1+ cell population depending on the STAT3/Bv8 signaling pathway. CAM-treated CD11b+Gr-1+ cells subsequently protect mice against LPS-induced shock mostly via increased IL-10, and protect mice from post-influenza pneumococcal pneumonia, mainly via decreased IFN-γ. (TIF) [file ppat.1006955.s017.tif]
